# Multimodal diffusion MRI biomarkers of white matter alterations and clinical impairment in mTBI

**DOI:** 10.3389/fnins.2026.1822384

**Published:** 2026-05-28

**Authors:** Maurizio Bergamino, Lauren R. Ott, Molly M. McElvogue, Ruchira M. Jha, Cindy Moreno, Ashley M. Stokes

**Affiliations:** 1 Barrow Neuroimaging Innovation Center, Barrow Neurological Institute, Phoenix, AZ, United States; 2 Departments of Neurology, Neurosurgery and Translational Science, Barrow Neurological Institute, Phoenix, AZ, United States

**Keywords:** concussion, diffusion kurtosis imaging, diffusion tensor imaging, free-water DTI, mild traumatic brain injury, neurite orientation dispersion and density imaging

## Abstract

**Introduction:**

Mild traumatic brain injury (mTBI), which includes concussion, often results in subtle white matter abnormalities that are not detectable with conventional neuroimaging. This study investigated microstructural brain changes in individuals with subacute mTBI using advanced diffusion MRI (dMRI) techniques, including multi-shell diffusion tensor imaging (DTI), free-water corrected DTI (fw-DTI), diffusion kurtosis imaging (DKI), and neurite orientation dispersion and density imaging (NODDI).

**Methods:**

Twenty individuals with mTBI and 24 healthy controls (HCs) underwent dMRI and cognitive assessment with the Montreal Cognitive Assessment (MoCA); mTBI participants were also evaluated using the Extended Glasgow Outcome Scale (GOS-E). Test-retest reliability was assessed in a subset of HCs.

**Results:**

Compared to controls, the mTBI group showed significantly lower MoCA scores and exhibited widespread reductions in fractional anisotropy (FA), mean kurtosis (MK), and kurtosis fractional anisotropy (KFA), along with increased free-water fraction and orientation dispersion indices. NODDI metrics revealed more localized reductions in neurite density (NDI), particularly in the genu of the corpus callosum, where lower NDI was associated with poorer cognitive performance. Elevated extracellular water content and greater neurite orientation dispersion were linked to worse functional and cognitive outcomes, even in areas without notable NDI loss, suggesting that early microenvironmental changes may influence recovery. In 11 HCs, test-retest analysis showed that NODDI and fw-DTI metrics had superior reliability compared to conventional DTI.

**Discussion:**

These findings support the utility of advanced, multi-parametric dMRI techniques for the sensitive detection of white matter alterations related to mTBI, highlighting their potential as reliable imaging biomarkers for clinical outcomes in mTBI.

## 1 Introduction

Mild traumatic brain injury (mTBI), which includes concussion, is a prevalent neurological condition characterized by transient disturbances in brain function that often lead to cognitive, behavioral, and neuropsychiatric symptoms. Despite its high incidence and substantial impact on daily functioning, conventional neuroimaging techniques such as computed tomography (CT) and standard magnetic resonance imaging (MRI) frequently fail to detect the subtle microstructural changes associated with mTBI (Guenette et al., 2018), particularly diffuse axonal injury that is a hallmark of this condition (Gonzalez et al., 2021; Rutgers et al., 2008; Khong et al., 2016).

Diffusion MRI (dMRI) is a powerful tool for investigating microstructural brain alterations. Standard diffusion tensor imaging (DTI), commonly used to assess white matter integrity, provides metrics like fractional anisotropy (FA), which reflects the directionality of water diffusion and is sensitive to white matter organization (Gonzalez et al., 2021; Khong et al., 2016; Alexander et al., 2007). Reduced fractional anisotropy (FA), frequently observed in individuals with mTBI, is a marker of underlying white matter disruption (Rutgers et al., 2008; Asken et al., 2018; Meier et al., 2016), and has been linked to cognitive impairment and symptom severity (Gonzalez et al., 2021).

However, DTI has several limitations. It assumes a single fiber orientation per voxel, which fails in regions with complex fiber configurations such as crossing, diverging, or converging tracts (Jones et al., 2013; Tournier et al., 2007). Additionally, partial volume effects (PVEs), where multiple tissue types contribute to a single voxel, can bias diffusion estimates, leading to inaccurate FA and mean diffusivity (MD) values, particularly in areas with small or heterogeneous structures (Bergamino et al., 2021; Pierpaoli et al., 1996).

To overcome these limitations, more advanced models such as free-water DTI (fw-DTI) (Pasternak et al., 2009; Hoy et al., 2014), diffusion kurtosis imaging (DKI) (Jensen et al., 2005), and neurite orientation dispersion and density imaging (NODDI) (Zhang et al., 2012; Huang et al., 2025) can provide enhanced sensitivity to microstructural complexity and offer more accurate characterizations of tissue changes.

The fw-DTI model was developed to separate tissue-specific diffusion from extracellular free water (e.g., cerebrospinal fluid or edema) using a bi-tensor model, which was originally applied to single b-value (i.e., single-shell) data (Pasternak et al., 2009). This approach improves upon standard DTI by reducing PVEs, allowing for more accurate diffusion metrics and estimation of the fw volume fraction (fw-F). However, estimating two compartments from single-shell data is mathematically ill-posed, with multiple solutions that can fit the data, and necessitating additional constraints or regularization that may introduce bias and limit sensitivity to subtle features (Yao et al., 2024; Correia et al., 2024; Bergamino et al., 2016).

In contrast, multi-shell fw-DTI uses multiple b-values to stabilize bi-tensor model fitting, enabling more robust and accurate estimation of tissue and free-water components (Hoy et al., 2014). This reduces reliance on regularization and improves reliability, especially in regions with high PVEs or complex microstructure. Multi-shell fw-DTI has shown greater biological validity in studies of neurodegenerative diseases such as Alzheimer’s disease, mild cognitive impairment (Bergamino et al., 2022; Dumont et al., 2019; Bergamino et al., 2021), and Parkinson’s disease (Ray et al., 2023; Bower et al., 2023). However, to date, very few studies have utilized multi-shell fw-dMRI to investigate mTBI.

DKI is an advanced dMRI technique that extends the standard DTI model by capturing the non-Gaussian nature of water diffusion in complex biological tissues (Jensen et al., 2005). Unlike DTI, which assumes Gaussian diffusion, DKI quantifies deviations from this behavior using kurtosis metrics, offering a more accurate depiction of microstructural complexity, particularly in regions with crossing fibers, high cellularity, or dense cytoarchitecture (Taha et al., 2022; Lin et al., 2018; Steven et al., 2014). This allows DKI to detect subtle pathological changes that may be missed by DTI, such as those in TBI or neurodegenerative disease (Alharshan et al., 2025; Steven et al., 2014).

DKI has been used to analyze brain changes in athletes after sport-related concussion, revealing alterations in kurtosis metrics during acute and subacute phases that tend to normalize with recovery (Muftuler et al., 2020). Other studies have reported DKI-based changes in white matter and the thalamus following mTBI, with kurtosis metrics providing complementary markers that may be more sensitive to post-concussion symptom burden than standard DTI measures (Karlsen et al., 2019). Moreover, these metrics have been associated with cognitive outcomes and overall symptom severity after concussion, highlighting their potential as biomarkers of subtle brain injury (Stenberg et al., 2021).

NODDI overcomes key limitations of standard DTI by offering more specific and biologically interpretable measures of brain microstructure (Zhang et al., 2012). Using a multicompartment model, NODDI separately estimates neurite density and orientation dispersion, enabling more accurate characterization of white matter especially in regions with complex fiber architecture. Studies have shown that NODDI provides greater specificity and can detect microstructural abnormalities that may be missed or mischaracterized by DTI (Huang et al., 2022; Timmers et al., 2016).

NODDI has been increasingly applied in concussion and mTBI research. For example, athletes with prior concussions have shown increased neurite density and reduced orientation dispersion, suggesting long-term microstructural reorganization (Churchill et al., 2017). Other studies have used NODDI to monitor recovery, demonstrating its enhanced sensitivity to subtle and persistent white matter changes beyond what standard DTI can reveal (Churchill et al., 2019).

In this study, we employed a multi-model dMRI framework to investigate microstructural alterations in individuals with mTBI. Diffusion metrics derived from standard multi-shell DTI, multi-shell fw-DTI, DKI, and NODDI were analyzed using voxel-wise group comparisons between mTBI subjects and healthy controls (HCs). Within the mTBI group, we also examined correlations between diffusion measures and clinical outcomes, including the Montreal Cognitive Assessment (MoCA) (Nasreddine et al., 2005) and the Extended Glasgow Outcome Scale (GOS-E) (Wilson et al., 2021). To evaluate reliability, test-retest reproducibility was assessed in a subset of 11 HCs using voxel-wise intra-class correlation coefficients (ICCs) (Shrout and Fleiss, 1979; Caceres et al., 2009) and Spearman’s rank correlation (ρ) (Yu and Hutson, 2024; Bere and Bjørkelund, 2009).

While standard DTI has long been important for mTBI research, it lacks the specificity to differentiate between distinct pathological processes such as edema and axonal disruption. By integrating fw-DTI, DKI, and NODDI, this study aims to achieve a more granular characterization of the subacute tissue environment. Overall, we seek to demonstrate the feasibility and added value of multi-parametric dMRI in detecting subtle brain changes associated with mTBI. Advanced models like DKI and NODDI offer enhanced sensitivity to complex tissue microstructure, providing a promising pathway toward the development of more robust and biologically meaningful imaging biomarkers for mTBI research.

## 2 Materials and methods

### 2.1 Subjects

A total of 24 HCs (12 females, mean age 29.4 ± 6.2 years) and 20 participants with mTBI (10 females, mean age 28.0 ± 7.5 years) were enrolled in this study. Most individuals in the mTBI group were diagnosed with concussion; however, two participants presented with intracranial hemorrhages (subdural and subarachnoid). For the assessment of test-retest reliability of the dMRI metrics, a subgroup of 11 HCs (4 females, mean age 31.8 ± 6.6 years) was utilized.

Participants in the mTBI group underwent the GOS-E assessment (Wilson et al., 1998), a structured interview designed to classify functional recovery following brain injury into one of eight categories, ranging from complete recovery to death. This tool provides a thorough evaluation of the individual’s independence and ability to perform everyday activities.

In addition, both HCs and mTBI participants completed the MoCA test, which measures cognitive abilities across several domains, such as attention, memory, executive function, language, visuospatial skills, and orientation. Previous studies have shown that individuals with concussion or TBI frequently score lower on the MoCA than HCs (Jafroudi et al., 2023; Kumar et al., 2015; de Guise et al., 2014). A complete summary of the demographic, clinical data, and motion parameters during the dMRI acquisition is shown in Table 1.

**TABLE 1:** Participant characteristics and statistical comparisons.

Group	# (F)	Age (SD)	MoCA	GOS-E	Mean days MRI exam after concussion
HC	24 (12)	29.4 (6.2)	28.47 (1.71)*	–	–
**Concussion**	20 (10)	28.0 (7.5)	25.05 (3.10)	6.4 (1.2)	10.73 (2.92)
Sex					
Chi-squared test	χ^2^ = 0.092; *p* = 0.762				
Age					
Shapiro-Wilk normality test					
HC		W = 0.912; *p* = 0.039	W = 0.817; *p* = 0.002		
Concussion		W = 0.883; *p* = 0.020	W = 0.898; *p* = 0.038		
Wilcoxon rank-sum		W = 274.5; *p* = 0.422	W = 347; p < 0.001		
**Motion during dMRI**	**ABS (mm)**	**REL (mm)**			
HC	0.590 (0.170)	0.251 (0.082)			
Concussion	0.651 (0.239)	0.248 (0.070)			
Shapiro-Wilk normality test					
HC	W = 0.901; *p* = 0.023	W = 0.902; *p* = 0.024			
Concussion	W = 0.968; *p* = 0.722	W = 0.963; *p* = 0.606			
Wilcoxon rank-sum	W = 194; *p* = 0.287	W = 229.5; *p* = 0.814			
Test-retest reliability					
**Group**	**# (F)**	**Age (SD)**	**MoCA**	**< Days between MRIs >**	
**HC**	11 (4)	31.8 (6.6)	29.36 (0.92)	14.1 (2.25) [11–18]	
* 20 HC with MoCA					

Group differences in sex distribution, age, Montreal Cognitive Assessment (MoCA), and MRI motion parameters during the dMRI sequences are summarized. Sex distribution was compared using a chi-squared test (). Age distributions were assessed for normality using the Shapiro-Wilk test and compared using a Wilcoxon rank-sum test. MRI motion metrics are shown as absolute (ABS, in mm) and relative (REL, in mm) displacements, with corresponding means and standard deviations. Shapiro-Wilk tests assessed normality of motion data in each group, and group comparisons were performed using Wilcoxon rank-sum tests. For the mTBI group, Glasgow Outcome Scale – Extended (GOS-E) scores and mean days between mTBI and MRI examination are also reported. Eleven HCs underwent repeat scans for test-retest reliability assessment.

*Only 20 HCs had MoCA score available. #: number of participants. F: Female. SD: Standard Deviation.

### 2.2 MRI acquisition

Diffusion MRI scans were conducted on a 3.0T Philips Ingenia system. Data acquisition utilized a two-dimensional echo-planar imaging (EPI) sequence with the following parameters: a field of view of 256 × 256 mm^2^, a matrix of 128 × 128, 72 slices, and an isotropic voxel size of 2.0 mm^3^. The repetition time (TR) was set to 7,000 ms, echo time (TE) to 101 ms, and the flip angle to 90°.

For the multi-shell protocol, one image without diffusion weighting (b = 0 s/mm^2^) was collected, along with four shells at b-values of 500, 1000, 2000, and 3000 s/mm^2^. Each shell included 20 diffusion directions, with phase encoding oriented in the anterior-posterior (A/P) direction. For EPI distortions corrections, an additional b = 0 s/mm^2^ image was acquired using reversed phase-encoding (posterior-anterior, P/A).

### 2.3 Data pre-processing

Diffusion MRI data were preprocessed using a combination of MRtrix3 (version 3.0.4-145) (Tournier et al., 2019), FSL (version 6.0.7.16) (Jenkinson et al., 2012), and the Advanced Normalization Tools (ANTs, version v2.4.4).^1^ The pipeline began with noise reduction via the dwidenoise function in MRtrix3 (Veraart et al., 2016), followed by correction for susceptibility-induced distortions, eddy currents, and subject motion using the TOPUP and eddy tools in FSL (Andersson and Sotiropoulos, 2016). Quality control of the diffusion data was performed using the eddy QC utilities to identify datasets with excessive artifacts (Bastiani et al., 2019). Participants were excluded if their scans contained a mean absolute head motion between volumes that exceeded 3 mm. A group-level brain template with 2 mm resolution was generated from all brain-extracted b0 images (by dwi2mask, Mrtrix3) (Dhollander et al., 2019) using the *antsMultivariateTemplateConstruction2.sh* script from the ANTs software. Standard DTI metrics were computed using dtifit (FSL). Free-water DTI, NODDI, and DKI metrics were computed as described in the subsequent subsections.

The ANTs Symmetric Normalization (SyN) algorithm (Avants et al., 2008) was used to co-register all diffusion MRI-derived maps to this common template space. Additionally, to further minimize anatomical variability across participants and enhance signal-to-noise ratio, all dMRI metrics in the group template space were smoothed using FSL’s fslmaths tool, applying an isotropic Gaussian kernel with a full width at half maximum (FWHM) of 4 mm. All statistical analyses were subsequently performed within this standardized group-wise template space. To identify the anatomical locations of significant clusters found in group comparisons and correlation analyses, the JHU DTI-based white matter atlases, including the ICBM-DTI-81 white-matter labels atlas and the JHU white-matter tractography atlas (Wakana et al., 2007; Hua et al., 2008), were used.

### 2.4 Multi-shell fw-DTI

Multi-shell free-water diffusion tensor imaging (fw-DTI) applies a two-compartment model to distinguish tissue-specific diffusion from extracellular free water, such as cerebrospinal fluid or edema. By utilizing diffusion data acquired at multiple b-values (multi-shell), this approach enhances the accuracy of estimating both tissue diffusion properties and the free water fraction. Hoy et al. showed that using two diffusion-weighted shells provides optimal parameter estimation, improving the reliability of FA measurements and minimizing errors from free water contamination (Hoy et al., 2014). In this study, fw-corrected diffusion metrics, including fw-FA and the free water index (fw-F), were computed using the DIPY library (version 1.10.0), specifically through the fwdti.FreeWaterTensorModel class and fwdtimodel.fit function (Garyfallidis et al., 2014),^2^ implemented in a custom Python script (version 3.11.5). The fw-DTI fitting process utilized b-values of 0, 500, 1,000, and 2,000 s/mm^2^ to estimate the fractional volume of the free-water compartment and the corrected diffusion tensor. This selection was made to provide a stable two-compartment fit while avoiding the higher-order non-Gaussian effects prevalent at higher b-values (Assaf et al., 2004).

Standard DTI metrics, particularly FA, reflect the directional coherence of water diffusion but lack specificity: reduced FA can result from axonal damage, demyelination, or increased extracellular water. fw-DTI explicitly separates the signal into tissue and extracellular compartments; the free-water fraction is thus specifically sensitive to vasogenic edema or neuroinflammation, both common in subacute mTBI.

### 2.5 Diffusion kurtosis imaging (DKI)

DKI was utilized to evaluate non-Gaussian diffusion characteristics in brain tissue, offering microstructural insights that extend beyond those provided by conventional DTI. In this study, three specific DKI metrics were used: mean kurtosis (MK), kurtosis fractional anisotropy (KFA), and mean kurtosis tensor (MKT). MK measures the overall deviation from Gaussian diffusion in all directions, making it sensitive to tissue complexity and microstructural integrity. KFA assesses the directional variation of kurtosis, providing enhanced contrast relative to standard FA, especially in areas with intricate fiber architecture where FA may be less effective. MKT serves as a summary metric, capturing the general kurtosis properties of the tissue and reflecting diffusion heterogeneity (Jensen et al., 2005). In the context of subacute mTBI, DKI metrics are expected to detect microstructural disruption with greater sensitivity than conventional DTI, as kurtosis is particularly responsive to changes in tissue barriers such as cell membranes and myelin, even when diffusion anisotropy remains relatively preserved.

All DKI-related metrics were derived using the DIPY software package. The diffusion kurtosis model was fitted to the data via the DiffusionKurtosisModel class, and the resulting MK, KFA, and MKT maps were generated using DIPY’s built-in functions.^3^ All processing was performed using a custom Python script, using all b-shells available.

### 2.6 Neurite orientation dispersion and density imaging (NODDI)

The NODDI model is an advanced diffusion MRI technique that enables a more nuanced assessment of brain microstructure by separating the diffusion signal into distinct tissue compartments (Zhang et al., 2012). With multi-shell diffusion MRI data, NODDI allows for the extraction of several important microstructural indices. The neurite density index (NDI) estimates the proportion of tissue volume occupied by axons and dendrites, providing a marker of neurite density. The orientation dispersion index (ODI) measures the degree of variability in neurite orientation, offering insight into the complexity of neural tissue organization. The isotropic volume fraction (FWF) quantifies the amount of freely diffusing water, which is particularly useful for identifying regions affected by CSF or neuroinflammatory processes. In the context of subacute mTBI, NODDI offers unique advantages over conventional DTI: NDI is expected to decrease specifically in regions with axonal loss or damage, while ODI may increase due to fiber disorganization or neuroinflammatory swelling, and FWF may rise in areas with vasogenic edema. Notably, NODDI can detect orientation dispersion changes even when neurite density is preserved, providing insight into early microstructural disorganization that precedes frank axonal loss.

For this study, NODDI parameter maps were generated using all b-shells available and a custom Python script based on the Accelerated Microstructure Imaging via Convex Optimization (AMICO, version 2.0.3) framework, which facilitates fast and reliable computation of NODDI metrics.^4^

### 2.7 Statistical analyses

Demographic and clinical characteristics for each group, including age, sex (as defined according to biological classification at birth), GOS-E scores, MoCA scores (available in all mTBI and 20 of 24 HCs), mean number of days from head injury to MRI examination, and head motion (absolute and relative) during dMRI acquisition are shown in Table 1. All values are reported as means ( ± standard deviation). Differences in sex distribution between groups were evaluated using a chi-square test. For age and head motion during dMRI scan, normality was assessed using the Shapiro–Wilk test; group comparisons were performed using the Wilcoxon rank-sum test.

The dMRI metrics analyzed in this study included: FA for standard DTI; fw-FA and fw-F for fw-DTI; KFA, MK, and MKT for DKI; FWF, ODI, and NDI for the NODDI model.

To evaluate test-retest reliability, a subset of 11 HCs underwent two MRI sessions spaced an average of 14.1 ± 2.25 days apart. Voxelwise ICCs were calculated for each diffusion metric using a two-way mixed-effects model for absolute agreement [ICC(3,1)], which is suitable for repeated measurements within the same subjects with fixed measurement sessions. ICC maps were generated to visualize the spatial distribution of reliability throughout the brain. Furthermore, we examined the distribution of voxelwise ICC values and reported the percentage of voxels exceeding thresholds of ICC > 0.50 (moderate reliability) and ICC > 0.75 (good reliability), along with the mean ± standard deviation of ICC values across the whole brain.

To further assess the test-retest reliability of diffusion-derived metrics, we also computed the Spearman rank correlation coefficient (ρ) between the first and second scan sessions for each HC subject. For each subject and each metric a voxelwise Spearman ρ map was computed. The mean and standard deviation of the average ρ values across subjects were then reported to quantify reproducibility. All test-retest reliability analyses were performed in R.^5^

Group-level differences in dMRI-derived metrics between HCs and participants with mTBI were examined using voxel-wise Student’s *t*-tests, implemented within a linear modeling approach in the group template space. Age, sex, and mean absolute head motion were incorporated as covariates. Additionally, for the mTBI group, voxel-wise associations between IVIM parameters and clinical assessments (MoCA and GOS-E scores) were analyzed using linear regression models, adjusting for the same set of covariates. All statistical computations were carried out using in-house R scripts.

To enhance sensitivity in voxel-based analyses and reduce reliance on arbitrary cluster thresholds, the Threshold-Free Cluster Enhancement (TFCE) technique was employed (Smith and Nichols, 2009). Multiple comparison correction was addressed by applying the Benjamini-Hochberg procedure for controlling the false discovery rate (FDR < 0.05), ensuring that the family-wise error rate did not exceed 0.05 (Benjamini and Hochberg, 1995).

To quantify the magnitude of group differences, Hedges’ g was calculated as the effect size (Hedges, 1981), with values of | g| greater than 0.93 interpreted as indicating a large effect (α = 0.05, power = 0.85). For voxel-wise correlation analyses, Spearman’s rank correlation coefficient (ρ) (Spearman, 1904) and the corresponding t statistics were reported. Correlation coefficients with an absolute value greater than 0.61 were considered to represent large effect sizes (α = 0.05, power = 0.85).

## 3 Results

There were no significant differences between the two groups in terms of sex distribution ( = 0.092, *p* = 0.762) or age (Wilcoxon *W* = 274.5, *p* = 0.42). However, a significant difference was observed in MoCA scores, with the mTBI group scoring lower than controls (Wilcoxon *W* = 347, *p* < 0.001). Within the mTBI group, the average GOS-E score was 6.4 ± 1.2, and the mean interval between injury and MRI scanning was 10.73 ± 2.92 days. Analysis of motion parameters derived from dMRI, including both absolute and relative motion, indicated no significant group differences (Wilcoxon *W* = 194, *p* = 0.287 for absolute motion; Wilcoxon *W* = 229.5, *p* = 0.814 for relative motion). All participants were included in the final analyses. A complete summary of all results and statistical analyses can be found in Table 1.

### 3.1 Test-retest reliability (ICC)

**FIGURE 1:**
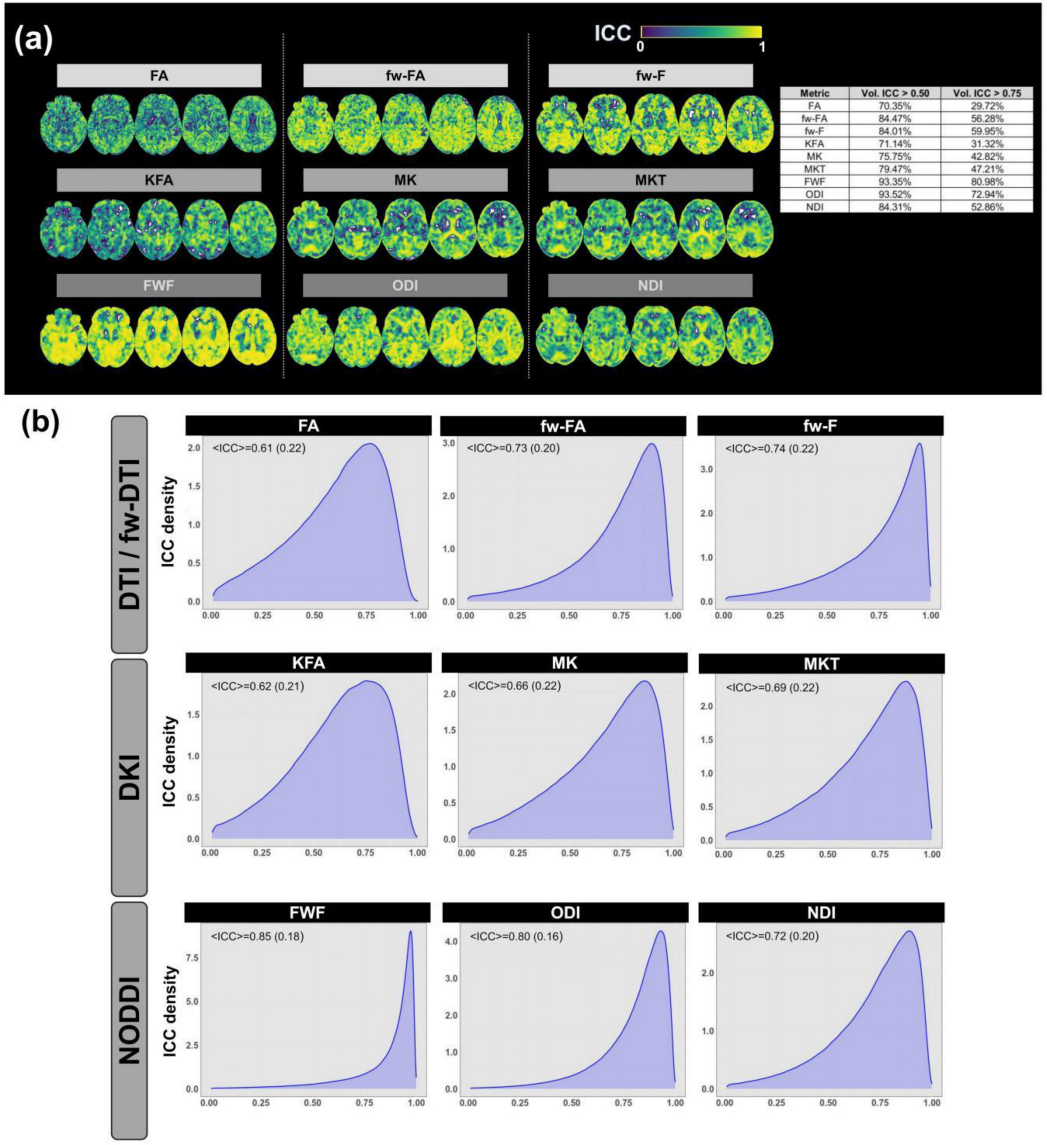
Test-retest reliability of dMRI-derived metrics (ICC). **(a)** Voxelwise intra-class correlation coefficient (ICC) maps for each dMRI metric across 11 healthy controls scanned twice. Maps are shown for FA (DTI), fw-FA and fw-F (fw-DTI), KFA, MK, and MKT (DKI), and FWF, ODI, and NDI (NODDI). Percentages of voxels showing moderate (ICC > 0.50) and good (ICC > 0.75) reliability are reported for each metric. **(b)** Distribution of voxelwise ICC values for each metric. The density plots summarize ICC reliability across the whole brain, with mean ICC values and standard deviations indicated in each panel. Metrics derived from fw-DTI and NODDI generally show higher reliability compared to standard DTI and DKI.

Figure 1 shows the voxelwise ICC maps and corresponding ICC density distributions for each diffusion metric. In Figure 1a, the ICC maps reveal varying degrees of test-retest reliability across different brain regions and metrics. Both fw-FA and fw-F demonstrated higher reliability (mean ICC: 0.73 ± 0.20 and 0.74 ± 0.22, respectively) than conventional DTI FA (mean ICC: 0.61 ± 0.22), with over 84% of voxels in these metrics exceeding an ICC threshold of 0.5 and more than half surpassing 0.75.

All DKI-derived metrics exhibited moderate to good reproducibility, with mean ICC values of 0.62 ± 0.21 for KFA, 0.66 ± 0.22 for MK, and 0.69 ± 0.22 for MKT. Each of these metrics had more than 70% of voxels with ICC values greater than 0.5.

NODDI metrics, particularly ODI and FWF, showed the strongest reliability overall (mean ICC: 0.80 ± 0.16 and 0.85 ± 0.18, respectively), with over 93% of voxels above the 0.5 ICC threshold and more than 70% exceeding 0.75.

Figure 1b displays the ICC density plots, which validate the voxelwise results; the FWF metric exhibited the highest density peak in the > 0.75 range, while standard FA and KFA demonstrated comparatively lower reliability.

### 3.2 Test-retest reliability (Spearman rank correlation coefficient)

**FIGURE 2:**
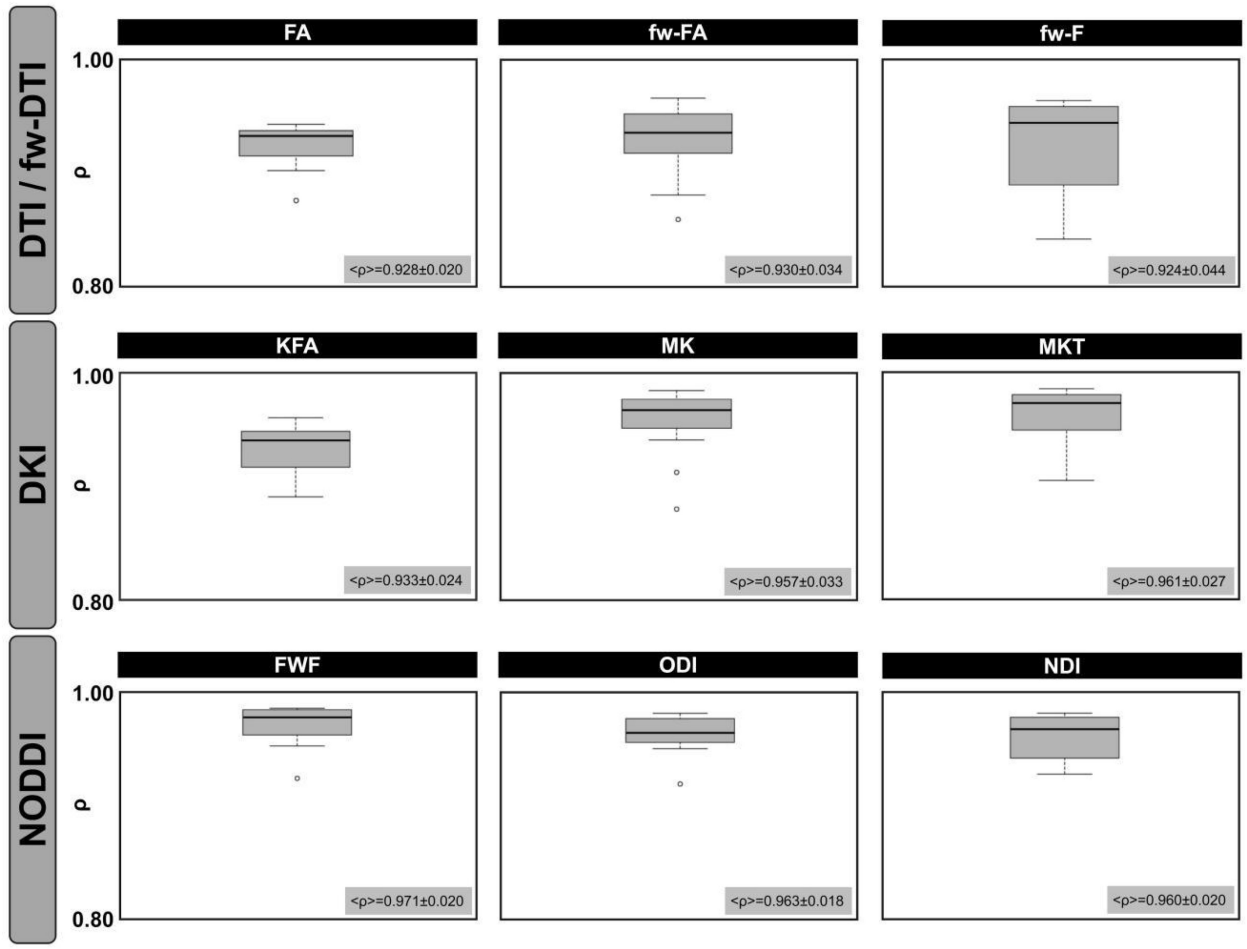
Test-retest reliability of dMRI metrics (Spearman’s rank correlation). Boxplots display the distribution of Spearman correlation coefficients (ρ) across 11 healthy control subjects for each diffusion MRI metric. Metrics are organized by model type: DTI and fw-DTI (top row), DKI (middle row), and NODDI (bottom row). Each boxplot represents the variability in subject-wise reliability across sessions and the interquartile range (25th–75th percentiles); The bold black line indicates the median. Mean Spearman’s ρ values and their standard deviations are reported in each panel, indicating high reproducibility for most metrics, particularly those derived from NODDI.

Figure 2 shows the test-retest reliability of the dMRI metrics in 11 HC subjects, as assessed by Spearman rank correlation coefficients (ρ). The boxplots illustrate the distribution of ρ values across individuals for each metric, grouped by model: DTI/fw-DTI (top row), DKI (middle row), and NODDI (bottom row). Among the DTI-derived measures, both FA and fw-FA demonstrated high reliability, with mean Spearman ρ values of 0.925 ± 0.020 and 0.930 ± 0.034, respectively. The fw-F metric also showed strong reproducibility (mean ρ = 0.924 ± 0.044). For the DKI metrics, KFA, MK, and MKT all exhibited robust test-retest performance, with mean ρ values of 0.933 ± 0.024, 0.957 ± 0.033, and 0.961 ± 0.027, respectively. The NODDI-derived parameters displayed the highest reliability overall, particularly for FWF, which achieved a mean ρ of 0.971 ± 0.020. ODI and NDI also showed excellent reproducibility, with mean ρ values of 0.963 ± 0.018 and 0.960 ± 0.020, respectively. The results indicate that all evaluated diffusion MRI metrics demonstrate good to excellent test-retest reliability, with NODDI and DKI measures generally outperforming standard DTI metrics.

**TABLE 2:** Group comparisons and clinical correlations using standard and free-water–corrected DTI metrics.

fw-DTI: fw-F															
	fw-F						fw-F-MoCA correlation						fw-F - GOS-E correlation		
	HC > Concussion			HC < Concussion			*t* > 0			*t* < 0			*t* < 0		
JHU white matter	Vol (%)	*t*	Max g	Vol (%)	*t*	Max g	Vol (%)	*t*	Max ρ	Vol (%)	*t*	Max ρ	Vol (%)	*t*	Max ρ
Superior longitudinal fasc R	0.41	3.814	1.187	–	–	–	–	–	–	0.80	−3.280	−0.693	12.10	−3.311	−0.819
Sup Longitudinal fasc temporal R	–	–	–	–	–	–	–	–	–	–	–	–	13.16	−3.248	−0.819
**ICBM-DTI 81**	**Vol (%)**	***t***	**Max g**	**Vol (%)**	***t***	**Max g**	**Vol (%)**	***t***	**Max ρ**	**Vol (%)**	***t***	**Max ρ**	**Vol (%)**	***t***	**Max ρ**
Pontine crossing tract	–	–	–	–	–	–	–	–	–	67.93	−4.282	−0.871	–	–	–
Splenium of corpus callosum	–	–	–	–	–	–	–	–	–	14.68	−3.877	−0.851	4.84	−3.030	−0.691
Corticospinal tract R	–	–	–	–	–	–	–	–	–	15.35	−3.296	−0.552	–	–	–
Corticospinal tract L	–	–	–	–	–	–	–	–	–	20.07	−3.860	−0.835	–	–	–
Medial lemniscus R	–	–	–	–	–	–	–	–	–	51.88	−4.150	−0.770	–	–	–
Medial lemniscus L	–	–	–	–	–	–	–	–	–	59.37	−4.088	−0.803	–	–	–
Inferior cerebellar peduncle R	–	–	–	–	–	–	–	–	–	29.65	−3.443	−0.740	–	–	–
Inferior cerebellar peduncle L	–	–	–	–	–	–	–	–	–	38.22	−4.058	−0.837	–	–	–
Superior cerebellar peduncle R	–	–	–	–	–	–	–	–	–	22.48	−3.677	−0.612	–	–	–
Cerebral peduncle R	–	–	–	–	–	–	–	–	–	18.61	−3.598	−0.808	–	–	–
Anterior corona radiata R	–	–	–	12.35	−3.889	−0.918	–	–	–	5.65	−3.702	−0.733	5.33	−2.878	−0.609
Superior corona radiata R	–	–	–	0.19	−3.415	−0.674	–	–	–	6.08	−3.637	−0.737	19.97	−2.993	−0.734
Superior corona radiata L	–	–	–	5.66	−3.042	−0.649	–	–	–	12.20	−3.074	−0.688	0.57	−2.918	−0.550
Posterior corona radiata R	–	–	–	–	–	–	–	–	–	3.27	−2.982	−0.539	28.92	−3.508	−0.854
Posterior corona radiata L	–	–	–	–	–	–	–	–	–	–	–	–	10.12	−2.827	−0.555
Fornix (cres)/Stria terminalis R	–	–	–	–	–	–	–	–	–	16.90	−3.968	−0.699	–	–	–
Superior longitudinal fasciculus R	–	–	–	–	–	–	–	–	–	1.83	−3.475	−0.667	13.05	−3.218	−0.819
Superior fronto-occipital fasciculus R	–	–	–	–	–	–	1.78	3.341	0.683	7.50	−3.104	−0.578	12.23	−2.614	−0.438
Superior fronto-occipital fasciculus L	–	–	–	21.50	−3.955	−0.975	–	–	–	–	–	–	–	–	–
Uncinate fasciculus L	–	–	–	–	–	–	–	–	–	19.41	−3.845	−0.722	–	–	–
Tapetum R	5.70	3.254	0.996	–	–	–	–	–	–	–	–	–	11.41	−3.103	−0.577
**DTI: FA**															
	**FA**			**FA-MoCA correlation**						**FA − GOS-E correlation**					
	**HC > Concussion**			**t > 0**			**t < 0**			**t > 0**					
**JHU white matter**	**Vol (%)**	**t**	**max g**	**Vol (%)**	**t**	**max ρ**	**Vol (%)**	**t**	**max ρ**	**Vol (%)**	**t**	**max ρ**			
Anterior thalamic radiation R	7.20	2.627	1.148	0.33	2.760	0.644	–	–	–	12.52	2.372	0.738			
Cingulum cingulate gyrus L	2.14	2.923	1.217	4.44	2.780	0.734	–	–	–	10.93	2.089	0.550			
Cingulum cingulate gyrus R	–	–	–	1.07	2.607	0.664	–	–	–	11.20	2.124	0.446			
Cingulum Hippo L	–	–	–	10.48	3.020	0.647	–	–	–	–	–	–			
Forceps Minor	4.00	2.885	1.412	1.42	2.664	0.545	0.40	−3.450	−0.686	20.58	2.268	0.669			
**ICBM-DTI 81**	**Vol (%)**	***t***	**Max g**	**Vol (%)**	***t***	**Max ρ**	**Vol (%)**	***t***	**Max ρ**	**Vol (%)**	***t***	**Max ρ**			
Genu of corpus callosum	2.90	2.480	0.913	5.40	2.725	0.545	–	–	–	18.60	2.584	0.452			
Body of corpus callosum	–	–	–	12.44	3.594	0.774	0.63	−3.382	−0.660	11.53	2.614	0.603			
Splenium of corpus callosum	–	–	–	26.11	3.181	0.802	1.46	−3.753	−0.712	5.42	2.537	0.496			
Inferior cerebellar peduncle R	–	–	–	22.21	3.272	0.777	–	–	–	–	–	–			
Superior cerebellar peduncle R	–	–	–	14.31	2.923	0.656	–	–	–	–	–	–			
Anterior corona radiata R	11.53	2.440	0.801	–	–	–	–	–	–	29.58	2.592	0.649			
Anterior corona radiata L	6.10	2.855	1.537	0.25	2.806	0.363	–	–	–	20.88	2.592	0.622			
Superior corona radiata R	10.39	2.638	0.979	5.89	3.509	0.828	–	–	–	16.08	2.751	0.672			
Posterior corona radiata R	1.85	3.129	1.325	11.78	2.932	0.664	–	–	–	38.25	3.071	0.686			
Posterior corona radiata L	–	–	–	6.11	2.690	0.649	–	–	–	24.77	3.016	0.656			
Posterior thalamic radiation L	3.47	2.942	1.279	10.16	3.275	0.713	0.40	−3.633	−0.731	2.94	2.903	0.419			
Sagittal stratum L	–	–	–	10.44	2.654	0.657	1.12	−3.792	−0.778	17.12	2.850	0.562			
External capsule R	4.49	2.260	0.751	–	–	–	–	–	–	12.60	2.458	0.445			
Cingulum (cingulate gyrus) R	–	–	–	1.62	3.317	0.749	–	–	–	14.65	2.633	0.393			
Cingulum (hippocampus) L	–	–	–	15.15	2.939	0.589	–	–	–	–	–	–			
Fornix (cres)/Stria terminalis L	–	–	–	17.24	2.805	0.647	–	–	–	8.00	2.876	0.396			
Superior longitudinal fasciculus R	1.01	2.351	0.813	0.77	2.723	0.599	–	–	–	10.58	2.575	0.637			
Superior longitudinal fasciculus L	0.32	2.922	1.275	3.45	2.678	0.677	–	–	–	15.06	2.890	0.752			
Superior fronto-occipital fasciculus R	14.60	2.273	0.782	–	–	–	–	–	–	–	–	–			

The table summarizes results for fractional anisotropy (FA), free-water corrected FA (fw-FA), and free-water index (fw-F) across three analyses: group comparisons (HC > mTBI and HC < mTBI), and correlations with MoCA and GOS-E scores. For each significant cluster (FDR < 0.05), the percentage of the white matter ROI volume (Vol %), mean *t*-value, and maximum effect-sizes (max g or max ρ) are reported. Only clusters occupying more than 10% of the respective white matter ROI in at least one analysis are listed. Johns Hopkins University (JHU) white matter atlas and the ICBM-DTI-81 atlas were used for this analysis. Full results, including clusters below 10%, are available in Supplementary Table 1.

### 3.3 Standard- and fw- DTI results

**FIGURE 3:**
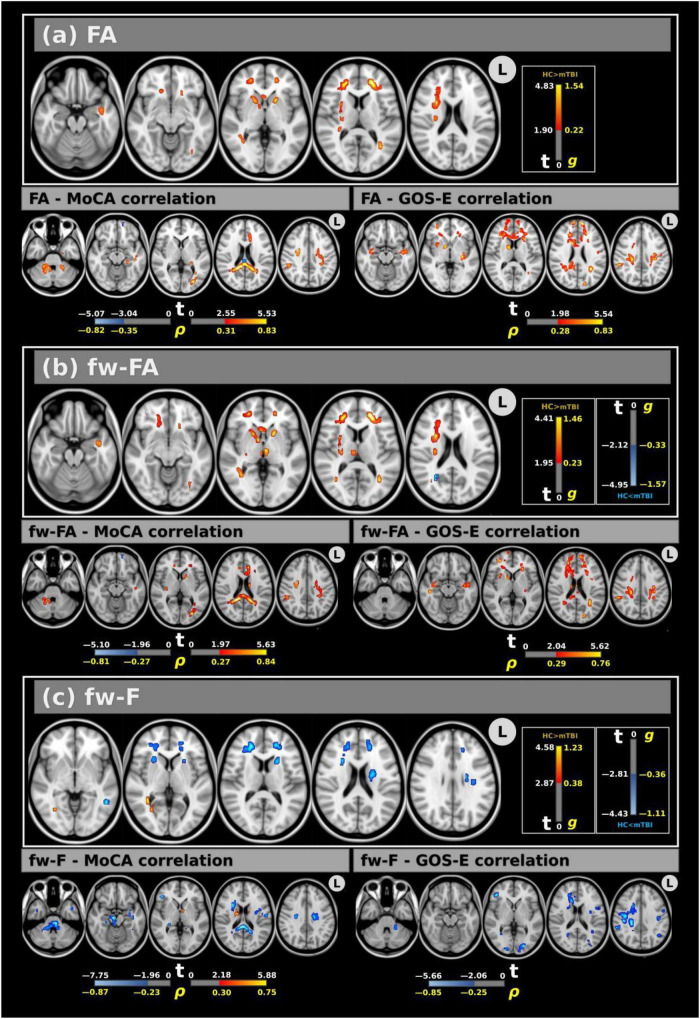
Voxel-based analysis for DTI/fw-DTI. Complete VBA analysis for the DTI **(a)** and fw-DTI **(b,c)** metrics. For each metric, group comparisons and correlations with MoCA and GOS-E score are displayed with *t*-values and effect-sizes. Significant clusters are shown after TFCE and FDR corrections (FDR < 0.05). g: effect-size and ρ: Spearman’s correlation coefficient. L: left.

Significant reductions in FA were observed across multiple white matter regions in the mTBI group compared to HCs as shown in Figure 3. The most pronounced clusters, characterized by moderate and large effect sizes, were identified in the right anterior corona radiata (*t* = 2.44), superior corona radiata (*t* = 2.64), and right superior fronto-occipital fasciculus (*t* = 2.27). Similarly, the fw-FA metric demonstrated significant decreases in the mTBI group, with large clusters localized to the right anterior limb of the internal capsule (*t* = 2.47), right anterior corona radiata (*t* = 2.21), superior corona radiata (*t* = 2.45), and right superior fronto-occipital fasciculus (*t* = 2.15). Effect size maps indicated moderate to large differences in these regions. Additionally, compared to HCs, the mTBI group exhibited significantly elevated fw-F values, particularly within the right anterior corona radiata (*t* = -3.89) and the left superior fronto-occipital fasciculus (*t* = -3.96), indicating increased extracellular free-water content in these areas.

In the mTBI group, lower FA and fw-FA values were significantly correlated with reduced MoCA scores in several white matter regions, indicating that microstructural disruption is associated with poorer cognitive performance. In alignment with these findings, higher fw-F was associated with lower MoCA scores, with large clusters in several white matter regions.

Both FA and fw-FA showed positive correlations with GOS-E scores, suggesting that higher white matter integrity is associated with better functional outcomes. Conversely, increased fw-F was negatively correlated with GOS-E, indicating that greater free-water content is associated with worse outcomes.

Table 2 presents a detailed summary of the results from both standard and fw–corrected DTI analyses, including cluster volume, mean t-values, and peak g-values and focusing specifically on clusters that occupy more than 10% of the corresponding white matter region in at least one group comparison or correlation analysis. Clusters surpassing this 10% volume threshold in any single analysis are shown in bold. A full list of all identified clusters, including those with volumes below 10%, is available in Supplementary Table 1.

### 3.4 DKI results

**FIGURE 4:**
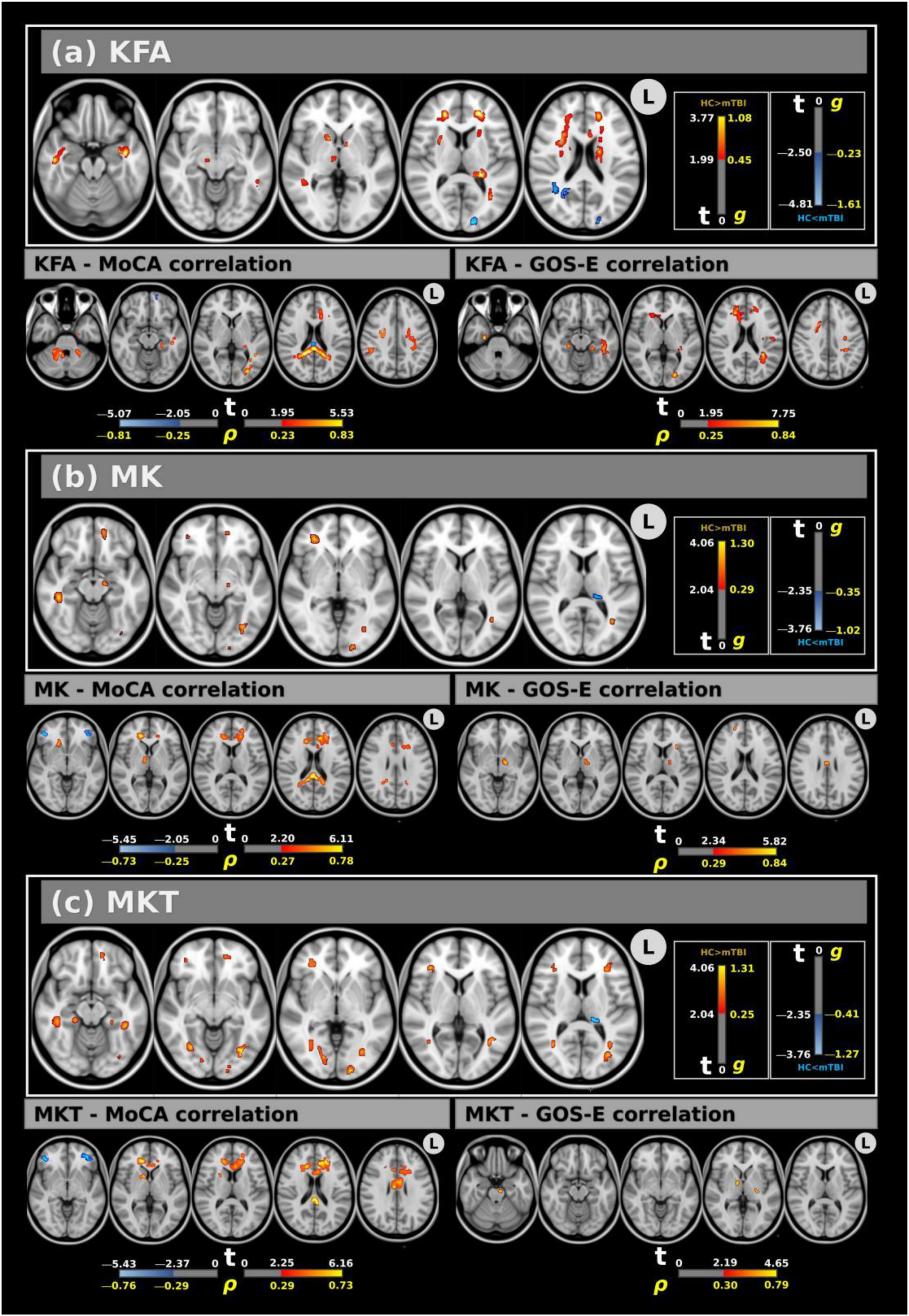
Voxel-based analysis for DKI metrics. Complete VBA analysis for DKI metrics **(a)** KFA, **(b)** MK, and **(c)** MKT. For each metric, groups comparisons and correlations with MoCA and GOS-E score are displayed with *t*-values and effect-sizes. Significant clusters are shown after TFCE and FDR corrections (FDR < 0.05). g: effect-size and ρ: Spearman’s correlation coefficient. L: left.

Figure 4 shows the voxel-based analysis results for DKI metrics. Individuals with mTBI exhibited lower DKI parameter values compared to the HCs. Significant group differences with large clusters were identified for KFA in several regions, including the middle cerebellar peduncle (*t* = 2.36), left anterior limb of the internal capsule (*t* = 2.45), left superior fronto-occipital fasciculus (*t* = 2.49), and left uncinate fasciculus (*t* = 2.39). For MK and MKT, only clusters with a relative volume below 10% were detected. Additionally, positive correlations between DKI metrics and GOS-E scores were observed, with large clusters (relative cluster volume > 10%) observed exclusively for KFA. Consistent with findings from DTI and fw-DTI analyses, reduced DKI values in the mTBI group were significantly associated with lower MoCA scores across multiple white matter regions, particularly within large clusters in the corpus callosum across all metrics.

Table 3 provides a detailed overview of the DKI analysis results, reporting cluster volumes, mean *t*-values, and peak g-values for clusters occupying more than 10% of the relevant white matter region in any group comparison or correlation analysis. Clusters exceeding this 10% volume threshold are highlighted in bold. A comprehensive list of all identified clusters, including those with volumes under 10%, can be found in Supplementary Table 2.

**TABLE 3:** Group comparisons and clinical correlations using DKI metrics.

DKI: KFA															
	KFA						KFA − MoCA correlation						KFA − GOS-E correlation		
	HC > Concussion			HC < Concussion			*t* > 0			*t* < 0			*t* > 0		
JHU white matter	Vol (%)	*t*	Max g	Vol (%)	*t*	Max g	Vol (%)	*t*	Max ρ	Vol (%)	*t*	Max ρ	Vol (%)	*t*	Max ρ
Cingulum cingulate gyrus R	0.93	2.577	0.666	**–**	**–**	**–**	1.07	2.172	0.664	**–**	**–**	**–**	10.32	2.765	0.691
Cingulum hippo L	4.83	2.935	0.591	**–**	**–**	**–**	10.48	2.516	0.647	**–**	**–**	**–**	9.09	3.822	0.807
Cingulum hippo R	–	–	–	**–**	**–**	**–**	**–**	**–**	**–**	**–**	**–**	**–**	13.87	3.832	0.791
**ICBM-DTI 81**	**Vol (%)**	***t***	**Max g**	**Vol (%)**	***t***	**Max g**	**Vol (%)**	***t***	**Max ρ**	**Vol (%)**	***t***	**Max ρ**	**Vol (%)**	***t***	**Max ρ**
Middle cerebellar peduncle	12.52	2.356	0.756	**–**	**–**	**–**	9.92	2.234	0.705	**–**	**–**	**–**	**–**	**–**	**–**
Genu of corpus callosum	0.80	2.623	0.469	**–**	**–**	**–**	5.40	2.021	0.545	**–**	**–**	**–**	10.61	3.237	0.614
Body of corpus callosum	0.34	2.323	0.486	**–**	**–**	**–**	12.44	2.161	0.674	0.63	−2.943	−0.660	5.57	2.958	0.564
Splenium of corpus callosum	0.20	2.757	0.736	0.61	−3.299	−1.422	26.11	2.651	0.802	1.46	−2.942	−0.712	**–**	**–**	**–**
Inferior cerebellar peduncle R	0.62	2.144	0.491	**–**	**–**	**–**	22.21	2.727	0.777	**–**	**–**	**–**	**–**	**–**	**–**
Superior cerebellar peduncle R	–	–	–	**–**	**–**	**–**	14.31	2.435	0.656	**–**	**–**	**–**	**–**	**–**	**–**
Anterior limb of internal capsule L	13.42	2.452	0.503	**–**	**–**	**–**	**–**	**–**	**–**	**–**	**–**	**–**	**–**	**–**	**–**
Anterior corona radiata R	11.39	2.677	1.072	**–**	**–**	**–**	**–**	**–**	**–**	**–**	**–**	**–**	11.67	3.183	0.669
Superior corona radiata R	5.64	2.501	0.839	**–**	**–**	**–**	5.89	2.924	0.828	**–**	**–**	**–**	11.72	3.493	0.721
Posterior corona radiata R	2.68	3.469	0.875	0.21	−3.292	−1.052	11.78	2.444	0.664	**–**	**–**	**–**	**–**	**–**	**–**
Posterior corona radiata L	–	–	–	**–**	**–**	**–**	6.11	2.242	0.649	**–**	**–**	**–**	14.40	3.413	0.765
Posterior thalamic radiation L	2.54	2.362	0.560	**–**	**–**	**–**	10.16	2.729	0.713	0.40	−3.117	−0.731	9.38	3.289	0.738
Sagittal stratum L	0.18	2.570	0.498	**–**	**–**	**–**	10.44	2.212	0.657	1.12	−3.117	−0.778	27.25	3.541	0.706
Cingulum (cingulate gyrus) R	–	–	–	**–**	**–**	**–**	1.62	2.764	0.749	**–**	**–**	**–**	10.67	2.971	0.628
Cingulum (hippocampus) R	–	–	–	**–**	**–**	**–**	**–**	**–**	**–**	**–**	**–**	**–**	22.17	4.256	0.791
Cingulum (hippocampus) L	–	–	–	**–**	**–**	**–**	15.15	2.449	0.589	**–**	**–**	**–**	15.32	4.200	0.807
Fornix (cres)/Stria terminalis R	–	–	–	**–**	**–**	**–**	**–**	**–**	**–**	**–**	**–**	**–**	20.91	3.942	0.791
Fornix (cres)/Stria terminalis L	3.02	2.502	0.471	**–**	**–**	**–**	17.24	2.337	0.647	**–**	**–**	**–**	17.51	3.812	0.746
Superior fronto-occipital fasciculus L	28.99	2.489	0.647	**–**	**–**	**–**	**–**	**–**	**–**	**–**	**–**	**–**	**–**	**–**	**–**
Uncinate fasciculus L	24.20	2.388	0.670	**–**	**–**	**–**	**–**	**–**	**–**	**–**	**–**	**–**	**–**	**–**	**–**
**DKI: MK**															
	**MK**						**MK − MoCA correlation**						**MK − GOS-E correlation**		
	**HC > Concussion**			**HC < Concussion**			**t > 0**			**t < 0**			**t > 0**		
**JHU white matter**	**Vol (%)**	***t***	**Max g**	**Vol (%)**	***t***	**Max g**	**Vol (%)**	***t***	**Max ρ**	**Vol (%)**	***t***	**Max ρ**	**Vol (%)**	***t***	**Max ρ**
Cingulum cingulate gyrus L	–	–	–	–	–	–	11.52	4.105	0.655	–	–	–	–	–	–
**ICBM-DTI 81**	**Vol (%)**	***t***	**Max g**	**Vol (%)**	***t***	**Max g**	**Vol (%)**	***t***	**Max ρ**	**Vol (%)**	***t***	**Max ρ**	**Vol (%)**	***t***	**Max ρ**
Genu of corpus callosum	–	–	–	–	–	–	11.41	4.148	0.695	–	–	–	–	–	–
Splenium of corpus callosum	–	–	–	–	–	–	13.22	4.105	0.775	–	–	–	–	–	–
Anterior corona radiata L	–	–	–	–	–	–	12.43	3.431	0.620	–	–	–	0.99	3.568	0.773
**DKI: MKT**															
	**MKT**						**MKT − MoCA correlation**						**MKT − GOS-E correlation**		
	**HC > Concussion**			**HC < Concussion**			**t > 0**			**t < 0**			**t > 0**		
**JHU white matter**	**Vol (%)**	***t***	**Max g**	**Vol (%)**	***t***	**Max g**	**Vol (%)**	***t***	**Max ρ**	**Vol (%)**	***t***	**Max ρ**	**Vol (%)**	***t***	**Max ρ**
Cingulum cingulate gyrus L	–	–	–	–	–	–	15.86	3.894	0.654	–	–	–	–	–	–
Forceps Minor	0.27	2.446	0.752	–	–	–	10.01	3.865	0.673	0.21	−3.103	−0.537	–	–	–
**ICBM-DTI 81**	**Vol (%)**	***t***	**Max g**	**Vol (%)**	***t***	**Max g**	**Vol (%)**	***t***	**Max ρ**	**Vol (%)**	***t***	**Max ρ**	**Vol (%)**	***t***	**Max ρ**
Genu of corpus callosum	–	–	–	–	–	–	22.18	3.770	0.673	–	–	–	–	–	–
Body of corpus callosum	–	–	–	–	–	–	14.10	3.644	0.601	–	–	–	–	–	–
Anterior corona radiata R	–	–	–	–	–	–	12.79	3.969	0.657	–	–	–	–	–	–
Anterior corona radiata L	–	–	–	–	–	–	15.19	3.728	0.633	–	–	–	–	–	–

The table summarizes results for kurtosis fractional anisotropy (KFA), mean kurtosis (MK), and mean kurtosis tensor (MKT) across three analyses: group comparisons (HC > mTBI and HC < mTBI), and correlations with MoCA and GOS-E scores. For each significant cluster (FDR < 0.05), the percentage of the white matter ROI volume (Vol %), mean t-value, and maximum effect-sizes (max g or max ρ) are reported. Only clusters occupying more than 10% of the respective white matter ROI in at least one analysis are listed. Johns Hopkins University (JHU) white matter atlas and the ICBM-DTI-81 atlas were used for this analysis. Full results, including clusters below 10%, are available in Supplementary Table 2.

### 3.5 NODDI results

**FIGURE 5:**
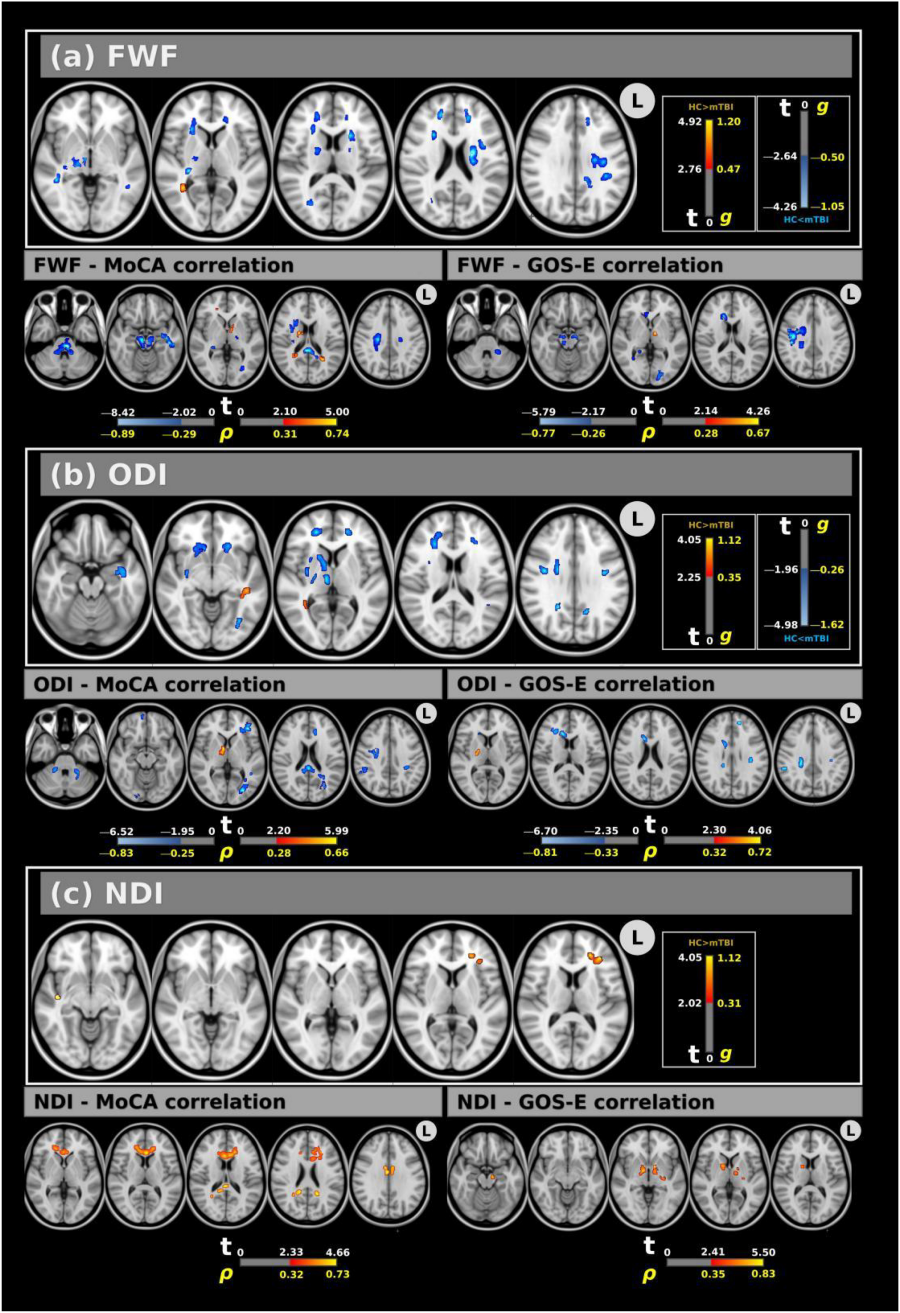
Voxel-based analysis for NODDI analysis. Complete VBA analysis for NODDI metrics **(a)** FWF, **(b)** ODI, and **(c)** NDI. For each metric, group comparisons and correlations with MoCA and GOS-E score are displayed with t-values and effect-sizes. Significant clusters are shown after TFCE and FDR corrections (FDR < 0.05). g: effect-size and ρ: Spearman’s correlation coefficient. L: left.

Figure 5 displays the results of the voxel-based analysis for NODDI metrics. In line with fw-F findings, individuals with mTBI exhibited significantly higher FWF values compared to the HC group. These differences were most prominent in large clusters within several white matter areas, including the right corticospinal tract (*t* = -2.79), right anterior corona radiata (*t* = -3.23), left superior corona radiata (*t* = -3.54), and left superior fronto-occipital fasciculus (*t* = -3.08). Similarly, ODI demonstrated elevated values in the mTBI group, with large effect-sizes observed in clusters within the right anterior limb of the internal capsule (*t* = -3.98), right superior corona radiata (*t* = -3.62), and right posterior corona radiata (*t* = -3.32). These results indicate widespread microstructural alterations in white matter related to mTBI. Additionally, several small clusters revealed lower NDI values in individuals with mTBI relative to controls, suggesting localized reductions in neurite density.

Correlation analyses further found significant correlations between NODDI metrics and MoCA. Both FWF and ODI exhibited significant negative correlations with MoCA scores in multiple white matter regions. Notably, NDI showed a robust positive correlation with MoCA within a large cluster in the genu of the corpus callosum. For GOS-E scores, significant negative correlations were observed for both FWF and ODI, while NDI demonstrated a significant positive correlation.

Table 4 shows a complete overview of the NODDI analysis results, reporting cluster volumes, mean *t*-values, and peak g-values for clusters occupying more than 10% of the relevant white matter region in any group comparison or correlation analysis. Clusters exceeding this 10% volume threshold are highlighted in bold. A comprehensive list of all identified clusters, including those with volumes under 10%, can be found in Supplementary Table 3.

### 3.6 Combined results

**FIGURE 6:**
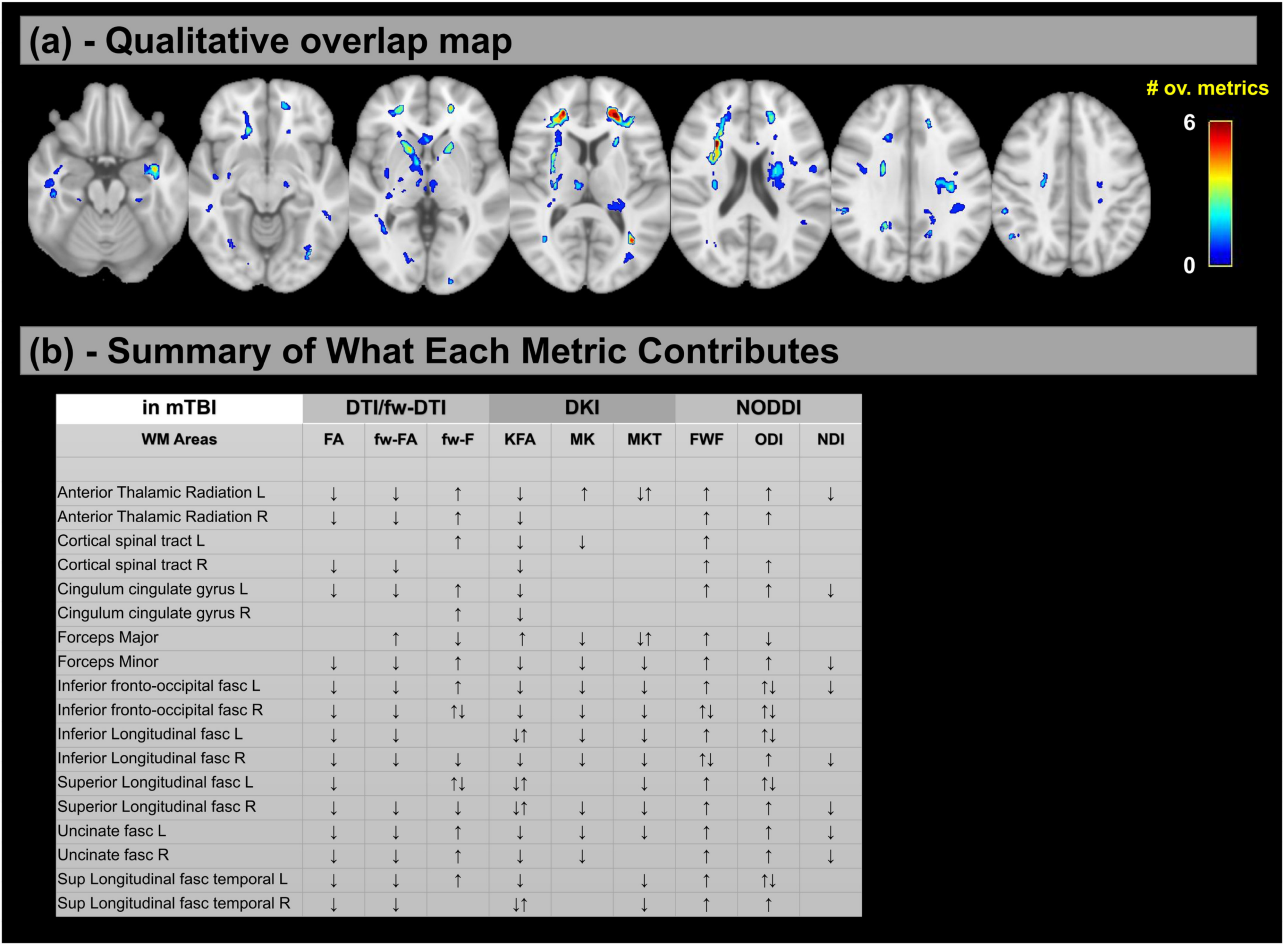
Combined diffusion MRI results across metrics. **(a)** Qualitative overlap map of white matter regions showing significant group differences. Color coding reflects the degree of overlap across metrics, with warmer colors indicating regions identified by a greater number of metrics. **(b)** Summary of metric-specific contributions. For each white matter tract, the table reports which diffusion metrics detect significant group differences, highlighting both shared and unique contributions across DTI/fw-DTI, DKI, and NODDI measures.

Figure 6 summarizes the combined results across all diffusion metrics. Panel (a) illustrates the spatial distribution of white matter regions showing significant group differences, with colors indicating the degree of overlap across metrics. Panel (b) provides a concise overview of the specific contribution of each metric, highlighting both shared and unique findings, as well as the white matter regions in which each metric detected significant group differences.

## 4 Discussion

This study aimed to detect and characterize white matter changes following mTBI, which often goes undetected by conventional neuroimaging. Using advanced dMRI techniques, such as multi-shell fw-DTI, DKI, and NODDI (Hoy et al., 2014; Jensen et al., 2005; Zhang et al., 2012), we sought to achieve a more sensitive and detailed assessment of microstructural brain alterations.

The central value of this multi-parametric approach is not simply that each metric independently detects abnormalities; rather, it lies in what the combination of metrics reveals that no single metric can. First, convergent evidence across biologically distinct metrics strengthens confidence in true tissue disruption. For example, when FA (DTI), MK (DKI), and NDI (NODDI) all decrease in the same region while FW (fw-DTI) increases, this convergent pattern provides multi-faceted evidence of axonal compromise with edema, far more compelling than any single metric alone. Second, and more importantly, the multi-parametric framework enables dissociation of qualitatively different pathological processes that standard DTI conflates under a single “reduced FA” umbrella. In our findings, some regions showed reduced NDI without elevated FW (suggesting predominant axonal loss), whereas other regions exhibited increased ODI and FW with preserved NDI (indicating early neurite disorganization and neuroinflammation without overt axonal loss). These distinct microenvironments would be indistinguishable using DTI alone yet may carry different prognostic implications. Third, different metrics correlated with different clinical outcomes: NDI loss was associated with cognitive performance (MoCA), whereas elevated FW and ODI were linked to functional recovery (GOS-E). This suggests that a composite multi-parametric signature may eventually allow for outcome-specific risk stratification. Thus, by integrating complementary metrics that capture different dimensions of tissue complexity, it is possible to obtain a more robust, biologically nuanced, and clinically informative picture of mTBI-related brain changes than any single technique could offer on its own.

In this study, the mTBI group showed lower MoCA scores than the control group, consistent with previous research indicating that cognitive difficulties are common after concussion or mTBI (Kumar et al., 2015). Furthermore, between-group differences in head motion during the dMRI scans were negligible, suggesting comparable levels of compliance across groups.

In our test–retest evaluation of 11 HCs, advanced dMRI metrics, especially those from NODDI and fw-DTI, showed strong reproducibility. NODDI indices (FWF, ODI, NDI) demonstrated the most robust reliability overall, with the majority of brain voxels exceeding accepted thresholds for good-to-excellent agreement on both ICCs and Spearman’s rank correlations. These results indicate that NODDI metrics (especially FWF and ODI) are stable and consistent across repeated acquisitions, underscoring their promise as sensitive biomarkers for detecting subtle microstructural change in clinical populations (Lehmann et al., 2021). fw-DTI metrics also outperformed conventional DTI measures, aligning with prior work showing that fw correction improves diffusion estimates by reducing partial volume (Bergamino et al., 2021). Lastly, DKI measures achieved moderate-to-good reliability, though generally below NODDI and fw-DTI metrics. Overall, the high test–retest reliability observed in this healthy cohort supports the use of NODDI and fw-DTI measures as valuable tools for probing white matter microstructure.

Our findings reveal marked microstructural alterations in the white matter of individuals with mTBI, as measured by both standard DTI and fw-DTI approaches. Specifically, we observed significant reductions in FA in several white matter areas, including the anterior and superior corona radiata and the superior fronto-occipital fasciculus. These differences were characterized by moderate to large effect sizes and were robustly recapitulated using fw-FA, which further highlights the vulnerability of these regions to brain injury.

These results align closely with previous dMRI studies reporting variation of FA in white matter following mTBI (Kou et al., 2010; Eierud et al., 2014). Reductions in FA are commonly interpreted as indicators of disruption to axonal membranes and myelin integrity (Shenton et al., 2012), and have been associated with post-concussive cognitive and functional impairments (Hulkower et al., 2013; Pavlovic et al., 2019). In this study, lower FA and fw-FA were significantly correlated with reduced MoCA and GOS-E scores, further supporting the idea that DTI-derived white matter changes may represent clinically meaningful injury (Kumar et al., 2015).

Importantly, the application of fw correction revealed additional insights through the fw index. The mTBI group showed significantly elevated fw-F values, indicative of increased extracellular fw content, in some white matter tracts such as the anterior corona radiata and superior fronto-occipital fasciculus. Several recent studies suggest that increased fw reflects neuroinflammatory processes, vasogenic edema, or axonal degeneration secondary to trauma (Vijayakumari et al., 2021; Pasternak et al., 2016).

Compared to standard FA, fw-FA demonstrated spatially overlapping and even more extensive regions of significant group difference, while also showing strengthened associations with clinical measures. This aligns with studies indicating that fw correction improves the biological specificity and robustness of DTI metrics, reducing partial volume effects (Hoy et al., 2014; Bergamino et al., 2021). Our results suggest that combining standard and fw-DTI metrics enhances the detection of mTBI-related microstructural changes and their links to cognitive and functional outcomes.

**TABLE 4:** Group comparisons and clinical correlations using NODDI metrics.

NODDI: FWF																		
	FWF						FWF − MoCA correlation						FWF − GOS-E correlation					
	HC > Concussion			HC < Concussion			*t* > 0			*t* < 0			*t* > 0			*t* < 0		
JHU white matter	Vol (%)	*t*	Max g	Vol (%)	*t*	Max g	Vol (%)	*t*	Max ρ	Vol (%)	*t*	Max ρ	Vol (%)	*t*	Max ρ	Vol (%)	*t*	Max ρ
Cortical spinal tract L	–	–	–	4.70	−3.199	−0.534	–	–	–	10.06	−4.423	−0.888	–	–	–	1.73	−3.501	−0.686
Cortical spinal tract R	–	–	–	2.89	−2.830	−0.661	–	–	–	13.55	−4.875	−0.695	–	–	–	2.75	−2.780	−0.647
**ICBM-DTI 81**	**Vol (%)**	**t**	**max g**	**Vol (%)**	**t**	**max g**	**Vol (%)**	**t**	**max ρ**	**Vol (%)**	**t**	**max ρ**	**Vol (%)**	**t**	**max ρ**	**Vol (%)**	**t**	**max ρ**
Middle cerebellar peduncle	–	–	–	2.60	−2.869	−0.651	–	–	–	11.28	−4.791	−0.769	–	–	–	2.28	−2.877	−0.471
Pontine crossing tract	–	–	–	2.07	−2.754	−0.565	–	–	–	57.60	−4.076	−0.875	–	–	–	–	–	–
Genu of corpus callosum	–	–	–	–	–	–	0.24	2.334	0.556	–	–	–	–	–	–	10.44	−3.649	−0.670
Corticospinal tract R	–	–	–	14.98	−2.792	−0.591	–	–	–	19.09	−4.508	−0.538	–	–	–	1.54	−2.537	−0.577
Corticospinal tract L	–	–	–	–	–	–	–	–	–	29.93	−4.096	−0.887	–	–	–	–	–	–
Medial lemniscus R	–	–	–	–	–	–	–	–	–	32.75	−3.294	−0.618	–	–	–	–	–	–
Medial lemniscus L	–	–	–	–	–	–	–	–	–	40.06	−3.799	−0.697	–	–	–	–	–	–
Inferior cerebellar peduncle R	–	–	–	0.72	−2.652	−0.570	–	–	–	14.57	−3.058	−0.645	–	–	–	–	–	–
Inferior cerebellar peduncle L	–	–	–	–	–	–	–	–	–	17.25	−4.841	−0.811	–	–	–	–	–	–
Superior cerebellar peduncle R	–	–	–	–	–	–	–	–	–	16.03	−4.875	−0.514	–	–	–	–	–	–
Cerebral peduncle R	–	–	–	2.02	−2.988	−0.606	–	–	–	15.80	−4.100	−0.705	–	–	–	7.42	−2.780	−0.594
Cerebral peduncle L	–	–	–	–	–	–	–	–	–	19.53	−3.472	−0.561	–	–	–	7.11	−3.372	−0.645
Anterior corona radiata R	–	–	–	12.73	−3.231	−0.698	3.99	2.606	0.635	1.99	−2.685	−0.570	–	–	–	0.76	−3.896	−0.614
Superior corona radiata R	–	–	–	–	–	–	–	–	–	19.47	−3.808	−0.587	–	–	–	15.23	−3.170	−0.706
Superior corona radiata L	–	–	–	15.78	−3.539	−0.534	–	–	–	10.78	−3.377	−0.689	–	–	–	–	–	–
Posterior corona radiata R	–	–	–	–	–	–	–	–	–	7.54	−3.674	−0.670	–	–	–	10.92	−2.694	−0.594
Fornix (cres)/Stria terminalis R	–	–	–	–	–	–	–	–	–	29.36	−4.008	−0.686	–	–	–	–	–	–
Fornix (cres)/Stria terminalis L	–	–	–	1.60	−3.006	−0.536	–	–	–	8.80	−2.957	−0.626	–	–	–	22.84	−3.236	−0.552
Superior fronto-occipital fasciculus L	–	–	–	13.02	−3.079	−0.571	–	–	–	0.99	−2.955	−0.380	–	–	–	–	–	–
Tapetum R	0.34	3.472	0.823	–	–	–	23.66	2.605	0.623	–	–	–	–	–	–	24.83	−3.487	−0.540
**NODDI: ODI**																		
	**ODI**						**ODI − MoCA correlation**						**ODI − GOS-E correlation**					
	**HC > Concussion**			**HC < Concussion**			***t* > 0**			***t* < 0**			***t* > 0**			***t* < 0**		
**ICBM-DTI 81**	**Vol (%)**	***t***	**Max g**	**Vol (%)**	***t***	**Max g**	**Vol (%)**	***t***	**Max ρ**	**Vol (%)**	***t***	**Max ρ**	**Vol (%)**	***t***	**Max ρ**	**Vol (%)**	***t***	**Max ρ**
Anterior limb of internal capsule R	–	–	–	21.61	−3.976	−1.121	1.40	3.405	0.635	–	–	–	–	–	–	–	–	–
Superior corona radiata R	–	–	–	11.67	−3.622	−0.949	–	–	–	5.33	−3.263	−0.646	–	–	–	1.92	−3.309	−0.741
Posterior corona radiata R	–	–	–	1.90	−3.323	−1.080	–	–	–	–	–	–	–	–	–	10.06	−3.620	−0.746
Superior fronto-occipital fasciculus R	–	–	–	14.00	−3.150	−0.812	–	–	–	–	–	–	–	–	–	–	–	–
**NODDI: NDI**																		
	**NDI**			**NDI − MoCA correlation**			**NDI − GOS-E correlation**											
	**HC > Concussion**			**t > 0**			**t > 0**											
**ICBM-DTI 81**	**Vol (%)**	***t***	**Max g**	**Vol (%)**	***t***	**Max ρ**	**Vol (%)**	***t***	**Max ρ**									
Genu of corpus callosum	–	–	–	30.37	2.893	0.707	–	–	–									
Anterior limb of internal capsule R	–	–	–	–	–	–	12.24	3.309	0.745									
Superior fronto-occipital fasciculus L	–	–	–	–	–	–	11.44	3.819	0.557									

The table summarizes results for fractional volume of isotropic water (FWF), orientation dispersion index (ODI), and neurite density index (NDI) across three analyses: group comparisons (HC > mTBI and HC < mTBI), and correlations with MoCA and GOS-E scores. For each significant cluster (FDR < 0.05), the percentage of the white matter ROI volume (Vol %), mean *t*-value, and maximum effect-sizes (max g or max ρ) are reported. Only clusters occupying more than 10% of the respective white matter ROI in at least one analysis are listed. Johns Hopkins University (JHU) white matter atlas and the ICBM-DTI-81 atlas were used for this analysis. Full results, including clusters below 10%, are available in Supplementary Table 3.

Voxelwise analysis revealed widespread reductions in diffusion kurtosis metrics, most prominently KFA, in participants with mTBI relative to HCs, with effects evident in major cerebellar-brainstem and association pathways (e.g., middle cerebellar peduncle, internal capsule, superior fronto-occipital fasciculus, uncinate fasciculus). Similar group-level abnormalities in DKI measures after mTBI have been reported previously, supporting the sensitivity of kurtosis-derived indices to microstructural disturbance (Karlsen et al., 2019; Stenberg et al., 2021).

Consistent with our findings, reduced KFA and lower MK have been described in concussed/mTBI cohorts, though the affected white matter regions vary across studies (Stokum et al., 2015; Stenberg et al., 2021). For example, Stokum et al. observed decreased radial kurtosis and MK in the anterior internal capsule across acute (∼10 days), 1-month, and 6-month time points, and decreased MK in the posterior internal capsule acutely; additionally, changes in MK from 1 to 6 months in the thalamus, internal capsule, and corpus callosum tracked with cognitive improvement (Stokum et al., 2015).

In our study cohort, lower DKI metrics were associated with poorer clinical status, specifically lower MoCA scores and worse GOS-E, supporting the clinical relevance of kurtosis-based diffusion measures in mTBI. The MoCA is a validated global cognitive screener across the TBI spectrum and provides a practical reference measure for imaging–cognition analyses (de Guise et al., 2014). Although direct relationships between DKI metrics and GOS-E have not been well characterized, prior work has linked kurtosis abnormalities to neuropsychological performance and symptom burden after mTBI; for example, Chung et al. reported that reduced axial kurtosis in the right superior longitudinal fasciculus was associated with poorer auditory-verbal working-memory performance within 4 weeks of injury (Chung et al., 2019).

The NODDI analyses revealed a consistent pattern of white matter alterations linked to mTBI, characterized by widespread increases in FWF and ODI, alongside more spatially confined decreases in NDI index. This pattern is in alignment with findings from other studies demonstrating that microstructural changes after mTBI are heterogeneous across tissue compartments rather than uniform (Palacios et al., 2020; Brett et al., 2024). Specifically, elevated FWF clusters in the mTBI group were identified across major projection pathways including the corticospinal tract and bilateral corona radiata, as well as association fibers such as the superior fronto-occipital fasciculus. These findings are consistent with reports of increased fw in white matter regions in mTBI cohorts (Gugger et al., 2023), supporting the interpretation that extracellular edema or neuroinflammation may underline these diffuse changes.

Correspondingly, increased ODI in overlapping locations, such as the anterior limb of the internal capsule and superior/posterior corona radiata, indicates a microstructural environment with greater angular dispersion of neurites. Similar increases in ODI have been documented following mTBI and are hypothesized to reflect axonal disorganization or compensatory reorganization in response to injury (Palacios et al., 2020; Oehr et al., 2021). The smaller, focal clusters of decreased NDI observed in this study may represent regions where axonal integrity or packing density is more profoundly reduced. This interpretation is consistent with prior diffusion imaging studies that found focal reductions in neurite density metrics corresponding to localized axonal injury after concussion (Churchill et al., 2019).

The timing of our assessment (mean 10.7 days post-injury) can coincide with a complex biological window where residual vasogenic edema may overlap with the peak of the neuroinflammatory response. Differentiating these two processes remains a significant challenge in neuroimaging; however, the integration of advanced diffusion models provides additional context. The increased fw-index in the fw-DTI model and the FWF in the NODDI framework may capture the expansion of the extracellular space in the mTBI group. This expansion is a common feature of both vasogenic edema (resulting from blood-brain barrier disruption) and inflammatory-related fluid accumulation. Meanwhile, metrics such as MK from DKI and NODDI-ODI are sensitive to structural complexity and restricted diffusion, and thus these metrics may more closely reflect the cellular components of neuroinflammation, such as microglial infiltration and astrogliosis. While these processes remain pathologically intertwined at this subacute stage, the multi-shell approach used here allows for a more comprehensive characterization of the tissue environment than conventional DTI.

Negative correlations between MoCA scores and both FWF and ODI indicate that greater apparent fw content and greater orientation dispersion are associated with poorer cognitive performance. Conversely, higher NDI, most notably within a sizable cluster in the genu of the corpus callosum, was linked to better MoCA outcomes. The genu is a major conduit for interhemispheric prefrontal connectivity; thus, preserved or higher neurite signal in this region may support cognitive domains that are sensitive to brain injury, including executive and attentional functions sampled by the MoCA subsections (Niogi and Mukherjee, 2010; Huang et al., 2005; Hofer and Frahm, 2006).

A similar pattern was observed with functional outcomes measured by the GOS-E. People with worse overall outcomes had higher FWF and ODI, while those with better outcomes had higher NDI. The fact that both cognitive tests (MoCA) and general functional measures (GOS-E) show similar links supports the idea that these brain imaging markers reflect real meaningful changes and not just random variation. Notably, FWF and ODI were related to poorer outcomes even in areas where NDI remained relatively stable. This suggests that early changes outside the neurons and how the brain fibers are organized may already affect function before there is significant loss of nerve fibers.

This study has several limitations. First, the imaging data for the mTBI group were collected at variable time points within a post-injury window of 7–15 days. Although this subacute phase is clinically important for observing evolving neurophysiological changes after mTBI, the variability in scan timing may have introduced heterogeneity in the imaging results. Additionally, the relatively small sample size and the heterogeneity within the mTBI group may reduce the statistical power and generalizability of the findings. Another final limitation relates to the use of the GOS-E as an outcome measure. While widely used and valuable, the GOS-E may lack the sensitivity to detect subtle but meaningful functional changes, especially in patients with milder impairments (Ranson et al., 2019). Consequently, it may occasionally misclassify or fail to capture individual differences in recovery trajectories.

Finally, this study included two participants in the mTBI group with evidence of intracranial hemorrhage. Because Susceptibility-Weighted Imaging (SWI) was not acquired, we could not systematically screen the entire cohort for the presence of microbleeds. The presence of blood degradation products can introduce local magnetic field inhomogeneities, which may affect the accuracy of diffusion-weighted signal modeling and the resulting indices. This factor should be considered when interpreting the results, and future studies would benefit from incorporating SWI to better characterize the impact of microhemorrhages on diffusion biomarkers.

In conclusion, advanced dMRI techniques such as NODDI, DKI, and fw-DTI effectively detect subtle white matter changes after mTBI that relate to cognitive and functional outcomes. These methods reveal complex alterations, including increased extracellular water and neurite dispersion alongside localized neurite density loss, that are not captured by traditional diffusion metrics alone. The association between these imaging markers and clinical measures like MoCA and GOS-E highlights their potential clinical relevance, particularly the importance of the genu of the corpus callosum in supporting cognitive function after injury. However, future research with larger, more uniform cohorts and additional outcome measures is needed to confirm these findings and explore the utility of advanced diffusion MRI biomarkers for prognosis and treatment monitoring in individuals with mTBI.

## Data Availability

The datasets presented in this article are not readily available because Data are unavailable due to privacy restrictions. Requests to access the datasets should be directed to maurizio.bergamino@barrowneuro.org.

## Footnotes

1
http://stnava.github.io/ANTs/

2
https://docs.dipy.org/stable/examples_built/reconstruction/reconst_fwdti.html

3
https://docs.dipy.org/stable/examples_built/reconstruction/reconst_dki.html

4
https://github.com/daducci/AMICO

5
https://www.r-project.org/
