# Supplementary material for: Multimodal diffusion MRI biomarkers of white matter alterations and clinical impairment in mTBI
Source: Front Neurosci. 2026 May 28;20:1822384. doi: 10.3389/fnins.2026.1822384 (PMC13254018; doi:10.3389/fnins.2026.1822384)
Supplement: Supplementary file 1 [file Table_1.docx]

**Sup. Table 1**

| **fw-DTI: fw-FA** | | | | | | | | | | | | | | | |
| --- | --- | --- | --- | --- | --- | --- | --- | --- | --- | --- | --- | --- | --- | --- | --- |
|  | **fw-FA** | | | | | | **fw-FA-MoCA correlation** | | | | | | **fw-FA - GOS-E correlation** | | |
|  | **HC > Concussion** | | | **HC < Concussion** | | | **t > 0** | | | **t < 0** | | | **t > 0** | | |
| **JHU white matter** | **Vol (%)** | **t** | **max g** | **Vol (%)** | **t** | **max g** | **Vol (%)** | **t** | **max ρ** | **Vol (%)** | **t** | **max ρ** | **Vol (%)** | **t** | **max ρ** |
| Anterior Thalamic Radiation L | 4.80 | 2.758 | 1.380 | — | — | — | 3.73 | 2.536 | 0.702 | 0.19 | -2.982 | -0.674 | 3.73 | 2.766 | 0.702 |
| Anterior Thalamic Radiation R | 9.11 | 2.454 | 1.393 | — | — | — | — | — | — | — | — | — | — | — | — |
| Cortical spinal tract L | — | — | — | — | — | — | 0.98 | 2.473 | 0.670 | — | — | — | 0.98 | 2.698 | 0.670 |
| Cortical spinal tract R | 1.70 | 2.258 | 0.804 | — | — | — | 1.22 | 2.849 | 0.826 | — | — | — | 1.22 | 3.108 | 0.826 |
| Cingulum cingulate gyrus L | 1.68 | 2.760 | 1.183 | — | — | — | 7.90 | 2.481 | 0.794 | — | — | — | 7.90 | 2.707 | 0.754 |
| Cingulum cingulate gyrus R | — | — | — | — | — | — | 2.82 | 2.449 | 0.639 | — | — | — | 2.82 | 2.671 | 0.639 |
| Forceps Major | — | — | — | 0.33 | -3.503 | -1.567 | 4.17 | 2.709 | 0.822 | — | — | — | 4.17 | 2.955 | 0.722 |
| Forceps Minor | 3.97 | 2.668 | 1.367 | — | — | — | 3.35 | 2.469 | 0.592 | — | — | — | 3.35 | 2.693 | 0.592 |
| Inferior fronto-occipital fasc L | 3.23 | 2.627 | 1.445 | — | — | — | 6.26 | 2.581 | 0.718 | 0.80 | -2.972 | -0.822 | 6.26 | 2.815 | 0.718 |
| Inferior fronto-occipital fasc R | 4.77 | 2.344 | 0.960 | — | — | — | 1.90 | 2.661 | 0.688 | — | — | — | 1.90 | 2.903 | 0.688 |
| Inferior Longitudinal fasc L | 1.89 | 2.705 | 1.458 | — | — | — | 5.18 | 2.598 | 0.718 | 0.69 | -3.108 | -0.822 | 5.18 | 2.834 | 0.718 |
| Inferior Longitudinal fasc R | 1.94 | 2.510 | 1.288 | — | — | — | — | — | — | — | — | — | — | — | — |
| Superior Longitudinal fasc L | — | — | — | — | — | — | 1.50 | 2.422 | 0.716 | — | — | — | 1.50 | 2.643 | 0.716 |
| Superior Longitudinal fasc R | 1.11 | 2.371 | 0.971 | — | — | — | 3.31 | 2.821 | 0.815 | — | — | — | 3.31 | 3.077 | 0.715 |
| Uncinate fasc L | 4.78 | 2.571 | 1.445 | — | — | — | 2.25 | 2.372 | 0.661 | 0.23 | -2.972 | -0.622 | 2.25 | 2.588 | 0.661 |
| Uncinate fasc R | 5.78 | 2.291 | 0.954 | — | — | — | — | — | — | — | — | — | — | — | — |
| Sup Longitudinal fasc temporal L | — | — | — | — | — | — | 1.32 | 2.415 | 0.685 | 0.15 | -3.192 | -0.808 | 1.32 | 2.635 | 0.685 |
| Sup Longitudinal fasc temporal R | 0.24 | 2.281 | 0.683 | — | — | — | 0.43 | 2.593 | 0.612 | — | — | — | 0.43 | 2.829 | 0.612 |
| **ICBM-DTI 81** | **Vol (%)** | **t** | **max g** | **Vol (%)** | **t** | **max g** | **Vol (%)** | **t** | **max ρ** | **Vol (%)** | **t** | **max ρ** | **Vol (%)** | **t** | **max ρ** |
| Middle cerebellar peduncle | — | — | — | — | — | — | 3.27 | 2.181 | 0.679 | — | — | — | 3.27 | 2.617 | 0.679 |
| Genu of corpus callosum | 5.76 | 2.501 | 1.296 | — | — | — | 9.41 | 2.074 | 0.592 | — | — | — | 9.41 | 2.489 | 0.592 |
| Body of corpus callosum | — | — | — | — | — | — | 18.75 | 2.133 | 0.746 | — | — | — | 18.75 | 2.560 | 0.675 |
| Splenium of corpus callosum | — | — | — | 0.10 | -3.412 | -1.466 | 26.39 | 2.506 | 0.822 | — | — | — | 26.39 | 3.007 | 0.722 |
| Inferior cerebellar peduncle R | — | — | — | — | — | — | 12.81 | 2.584 | 0.743 | — | — | — | 12.81 | 3.101 | 0.743 |
| Superior cerebellar peduncle R | — | — | — | — | — | — | 8.37 | 2.346 | 0.610 | — | — | — | 8.37 | 2.816 | 0.610 |
| Anterior limb of internal capsule R | 12.17 | 2.470 | 1.393 | — | — | — | — | — | — | — | — | — | — | — | — |
| Anterior limb of internal capsule L | 6.36 | 2.931 | 1.313 | — | — | — | 2.85 | 2.309 | 0.483 | — | — | — | 2.85 | 2.771 | 0.483 |
| Posterior limb of internal capsule R | 3.84 | 2.411 | 0.901 | — | — | — | — | — | — | — | — | — | — | — | — |
| Retrolenticular part of internal capsule R | 0.87 | 2.170 | 0.795 | — | — | — | 1.39 | 2.363 | 0.636 | — | — | — | 1.39 | 2.836 | 0.636 |
| Anterior corona radiata R | 12.86 | 2.210 | 0.894 | — | — | — | 0.50 | 2.317 | 0.577 | — | — | — | 0.50 | 2.780 | 0.577 |
| Anterior corona radiata L | 7.14 | 2.683 | 1.441 | — | — | — | 7.95 | 2.107 | 0.661 | — | — | — | 7.95 | 2.529 | 0.661 |
| Superior corona radiata R | 15.04 | 2.448 | 0.999 | — | — | — | 5.64 | 2.943 | 0.835 | — | — | — | 5.64 | 3.531 | 0.735 |
| Superior corona radiata L | — | — | — | — | — | — | 5.85 | 2.255 | 0.670 | — | — | — | 5.85 | 2.706 | 0.670 |
| Posterior corona radiata R | — | — | — | 0.30 | -3.383 | -0.992 | 13.09 | 2.407 | 0.674 | — | — | — | 13.09 | 2.889 | 0.674 |
| Posterior corona radiata L | — | — | — | — | — | — | 7.16 | 2.261 | 0.675 | — | — | — | 7.16 | 2.714 | 0.675 |
| Posterior thalamic radiation R | 7.83 | 2.524 | 1.274 | 0.43 | -3.420 | -1.246 | 0.96 | 2.003 | 0.606 | — | — | — | 0.96 | 2.403 | 0.606 |
| Posterior thalamic radiation L | 2.92 | 2.800 | 1.301 | — | — | — | 14.53 | 2.472 | 0.718 | 0.33 | -3.182 | -0.727 | 14.53 | 2.966 | 0.718 |
| Sagittal stratum R | 0.63 | 2.383 | 0.881 | — | — | — | 0.27 | 2.360 | 0.618 | — | — | — | 0.27 | 2.831 | 0.618 |
| Sagittal stratum L | — | — | — | — | — | — | 4.26 | 2.222 | 0.601 | 0.99 | -3.139 | -0.781 | 4.26 | 2.666 | 0.601 |
| External capsule R | 5.51 | 2.143 | 0.796 | — | — | — | 4.85 | 2.431 | 0.647 | — | — | — | 4.85 | 2.917 | 0.647 |
| External capsule L | — | — | — | — | — | — | 0.77 | 2.173 | 0.576 | — | — | — | 0.77 | 2.608 | 0.576 |
| Cingulum (cingulate gyrus) R | — | — | — | — | — | — | 1.41 | 2.602 | 0.705 | — | — | — | 1.41 | 3.122 | 0.705 |
| Cingulum (cingulate gyrus) L | — | — | — | — | — | — | 0.65 | 1.855 | 0.486 | — | — | — | 0.65 | 2.226 | 0.486 |
| Fornix (cres) / Stria terminalis L | — | — | — | — | — | — | 1.16 | 2.194 | 0.547 | — | — | — | 1.16 | 2.633 | 0.547 |
| Superior longitudinal fasciculus R | 1.04 | 2.299 | 0.850 | — | — | — | 1.01 | 2.049 | 0.593 | — | — | — | 1.01 | 2.459 | 0.593 |
| Superior longitudinal fasciculus L | — | — | — | — | — | — | 4.54 | 2.189 | 0.681 | — | — | — | 4.54 | 2.627 | 0.681 |
| Superior fronto-occipital fasciculus R | 22.09 | 2.154 | 0.732 | — | — | — | — | — | — | — | — | — | — | — | — |
| Uncinate fasciculus L | 2.13 | 2.476 | 1.267 | — | — | — | — | — | — | — | — | — | — | — | — |
| Tapetum R | — | — | — | — | — | — | 5.03 | 2.096 | 0.618 | — | — | — | 5.03 | 2.515 | 0.618 |
| Tapetum L | — | — | — | — | — | — | 3.00 | 2.133 | 0.628 | — | — | — | 3.00 | 2.560 | 0.628 |
|  |  |  |  |  |  |  |  |  |  |  |  |  |  |  |  |
| **fw-DTI: fw-F** | | | | | | | | | | | | | | | |
|  | **fw-F** | | | | | | **fw-F-MoCA correlation** | | | | | | **fw-F - GOS-E correlation** | | |
|  | **HC > Concussion** | | | **HC < Concussion** | | | **t > 0** | | | **t < 0** | | | **t < 0** | | |
| **JHU white matter** | **Vol (%)** | **t** | **max g** | **Vol (%)** | **t** | **max g** | **Vol (%)** | **t** | **max ρ** | **Vol (%)** | **t** | **max ρ** | **Vol (%)** | **t** | **max ρ** |
| Anterior Thalamic Radiation L | — | — | — | 2.65 | -3.491 | -0.870 | 1.36 | 3.340 | 0.700 | 1.59 | -3.578 | -0.787 | 0.36 | -2.802 | -0.498 |
| Anterior Thalamic Radiation R | — | — | — | 4.56 | -4.016 | -0.939 | 1.21 | 3.651 | 0.710 | 6.90 | -3.481 | -0.808 | 1.19 | -2.789 | -0.593 |
| Cortical spinal tract L | — | — | — | 0.51 | -3.478 | -0.844 | — | — | — | 7.38 | -3.340 | -0.871 | 0.38 | -3.152 | -0.480 |
| Cortical spinal tract R | — | — | — | — | — | — | — | — | — | 7.20 | -3.168 | -0.766 | 4.17 | -3.502 | -0.845 |
| Cingulum cingulate gyrus L | — | — | — | 4.18 | -3.675 | -0.941 | — | — | — | 1.17 | -3.158 | -0.710 | — | — | — |
| Cingulum cingulate gyrus R | — | — | — | 0.54 | -3.669 | -1.108 | — | — | — | 1.63 | -3.171 | -0.667 | 1.38 | -2.957 | -0.674 |
| Cingulum Hippo R | — | — | — | — | — | — | — | — | — | 4.90 | -3.481 | -0.639 | — | — | — |
| Forceps Major | 0.22 | 3.111 | 0.910 | — | — | — | — | — | — | 2.80 | -3.018 | -0.710 | 5.57 | -3.356 | -0.783 |
| Forceps Minor | — | — | — | 4.61 | -4.016 | -1.108 | — | — | — | — | — | — | 2.14 | -3.110 | -0.674 |
| Inferior fronto-occipital fasc L | — | — | — | 0.63 | -3.347 | -0.635 | — | — | — | 0.73 | -3.298 | -0.705 | 1.38 | -3.348 | -0.780 |
| Inferior fronto-occipital fasc R | 0.84 | 3.380 | 1.203 | 1.98 | -3.391 | -0.725 | — | — | — | 1.50 | -3.293 | -0.702 | 2.72 | -3.565 | -0.812 |
| Inferior Longitudinal fasc L | — | — | — | — | — | — | — | — | — | 3.34 | -3.383 | -0.777 | 1.84 | -3.249 | -0.780 |
| Inferior Longitudinal fasc R | 0.34 | 3.306 | 1.188 | — | — | — | — | — | — | 1.96 | -3.658 | -0.771 | — | — | — |
| Superior Longitudinal fasc L | 0.34 | 3.664 | 1.221 | 0.82 | -4.127 | -0.986 | — | — | — | 3.93 | -3.092 | -0.777 | 8.04 | -3.171 | -0.764 |
| Superior Longitudinal fasc R | 0.41 | 3.814 | 1.187 | — | — | — | — | — | — | 0.80 | -3.280 | -0.693 | 12.10 | -3.311 | -0.819 |
| Uncinate fasc L | — | — | — | 0.38 | -3.173 | -0.631 | — | — | — | 2.23 | -3.198 | -0.738 | — | — | — |
| Uncinate fasc R | — | — | — | 0.28 | -3.399 | -0.754 | — | — | — | — | — | — | 0.22 | -4.359 | -0.738 |
| Sup Longitudinal fasc temporal L | — | — | — | 1.65 | -3.427 | -0.986 | — | — | — | 5.69 | -3.205 | -0.777 | 8.58 | -3.092 | -0.745 |
| Sup Longitudinal fasc temporal R | — | — | — | — | — | — | — | — | — | — | — | — | 13.16 | -3.248 | -0.819 |
| **ICBM-DTI 81** | **Vol (%)** | **t** | **max g** | **Vol (%)** | **t** | **max g** | **Vol (%)** | **t** | **max ρ** | **Vol (%)** | **t** | **max ρ** | **Vol (%)** | **t** | **max ρ** |
| Middle cerebellar peduncle | — | — | — | — | — | — | — | — | — | 9.31 | -3.406 | -0.806 | 2.01 | -3.206 | -0.557 |
| Pontine crossing tract | — | — | — | — | — | — | — | — | — | 67.93 | -4.282 | -0.871 | — | — | — |
| Genu of corpus callosum | — | — | — | — | — | — | — | — | — | — | — | — | 7.28 | -3.298 | -0.652 |
| Body of corpus callosum | — | — | — | 0.16 | -3.334 | -0.533 | — | — | — | 2.84 | -3.466 | -0.607 | 4.68 | -2.931 | -0.590 |
| Splenium of corpus callosum | — | — | — | — | — | — | — | — | — | 14.68 | -3.877 | -0.851 | 4.84 | -3.030 | -0.691 |
| Corticospinal tract R | — | — | — | — | — | — | — | — | — | 15.35 | -3.296 | -0.552 | — | — | — |
| Corticospinal tract L | — | — | — | — | — | — | — | — | — | 20.07 | -3.860 | -0.835 | — | — | — |
| Medial lemniscus R | — | — | — | — | — | — | — | — | — | 51.88 | -4.150 | -0.770 | — | — | — |
| Medial lemniscus L | — | — | — | — | — | — | — | — | — | 59.37 | -4.088 | -0.803 | — | — | — |
| Inferior cerebellar peduncle R | — | — | — | — | — | — | — | — | — | 29.65 | -3.443 | -0.740 | — | — | — |
| Inferior cerebellar peduncle L | — | — | — | — | — | — | — | — | — | 38.22 | -4.058 | -0.837 | — | — | — |
| Superior cerebellar peduncle R | — | — | — | — | — | — | — | — | — | 22.48 | -3.677 | -0.612 | — | — | — |
| Cerebral peduncle R | — | — | — | — | — | — | — | — | — | 18.61 | -3.598 | -0.808 | — | — | — |
| Anterior limb of internal capsule R | — | — | — | 0.70 | -3.327 | -0.687 | 1.72 | 3.381 | 0.691 | 1.21 | -3.238 | -0.635 | — | — | — |
| Anterior limb of internal capsule L | — | — | — | 8.91 | -3.415 | -0.730 | 0.76 | 3.229 | 0.700 | — | — | — | — | — | — |
| Posterior limb of internal capsule R | — | — | — | — | — | — | 3.84 | 3.461 | 0.745 | — | — | — | — | — | — |
| Posterior limb of internal capsule L | — | — | — | 1.23 | -3.982 | -0.859 | — | — | — | — | — | — | — | — | — |
| Anterior corona radiata R | — | — | — | 12.35 | -3.889 | -0.918 | — | — | — | 5.65 | -3.702 | -0.733 | 5.33 | -2.878 | -0.609 |
| Anterior corona radiata L | — | — | — | 4.14 | -3.148 | -0.583 | — | — | — | — | — | — | — | — | — |
| Superior corona radiata R | — | — | — | 0.19 | -3.415 | -0.674 | — | — | — | 6.08 | -3.637 | -0.737 | 19.97 | -2.993 | -0.734 |
| Superior corona radiata L | — | — | — | 5.66 | -3.042 | -0.649 | — | — | — | 12.20 | -3.074 | -0.688 | 0.57 | -2.918 | -0.550 |
| Posterior corona radiata R | — | — | — | — | — | — | — | — | — | 3.27 | -2.982 | -0.539 | 28.92 | -3.508 | -0.854 |
| Posterior corona radiata L | — | — | — | — | — | — | — | — | — | — | — | — | 10.12 | -2.827 | -0.555 |
| Posterior thalamic radiation R | 4.03 | 3.405 | 1.203 | — | — | — | — | — | — | — | — | — | 2.97 | -3.154 | -0.616 |
| Posterior thalamic radiation L | — | — | — | — | — | — | — | — | — | 1.13 | -3.296 | -0.483 | — | — | — |
| Sagittal stratum L | — | — | — | — | — | — | — | — | — | 1.26 | -3.431 | -0.641 | — | — | — |
| External capsule R | — | — | — | 1.98 | -3.860 | -0.971 | — | — | — | 0.91 | -3.311 | -0.638 | — | — | — |
| External capsule L | — | — | — | 0.47 | -3.998 | -0.888 | — | — | — | 2.42 | -3.648 | -0.738 | — | — | — |
| Cingulum (cingulate gyrus) R | — | — | — | — | — | — | — | — | — | 2.13 | -3.395 | -0.672 | 1.75 | -3.125 | -0.674 |
| Cingulum (cingulate gyrus) L | — | — | — | 0.95 | -3.287 | -0.623 | — | — | — | 2.54 | -3.717 | -0.710 | — | — | — |
| Cingulum (hippocampus) R | — | — | — | — | — | — | — | — | — | 9.22 | -3.623 | -0.639 | — | — | — |
| Fornix (cres) / Stria terminalis R | — | — | — | — | — | — | — | — | — | 16.90 | -3.968 | -0.699 | — | — | — |
| Fornix (cres) / Stria terminalis L | — | — | — | — | — | — | — | — | — | 4.98 | -3.424 | -0.549 | — | — | — |
| Superior longitudinal fasciculus R | — | — | — | — | — | — | — | — | — | 1.83 | -3.475 | -0.667 | 13.05 | -3.218 | -0.819 |
| Superior longitudinal fasciculus L | — | — | — | 1.82 | -3.573 | -0.891 | — | — | — | 4.53 | -2.905 | -0.580 | 7.81 | -3.014 | -0.745 |
| Superior fronto-occipital fasciculus R | — | — | — | — | — | — | 1.78 | 3.341 | 0.683 | 7.50 | -3.104 | -0.578 | 12.23 | -2.614 | -0.438 |
| Superior fronto-occipital fasciculus L | — | — | — | 21.50 | -3.955 | -0.975 | — | — | — | — | — | — | — | — | — |
| Uncinate fasciculus L | — | — | — | — | — | — | — | — | — | 19.41 | -3.845 | -0.722 | — | — | — |
| Tapetum R | 5.70 | 3.254 | 0.996 | — | — | — | — | — | — | — | — | — | 11.41 | -3.103 | -0.577 |
|  |  |  |  |  |  |  |  |  |  |  |  |  |  |  |  |
| **DTI: FA** | | | | | | | | | | | | |  |  |  |
|  | **FA** | | | **FA-MoCA correlation** | | | | | | **FA - GOS-E correlation** | | |  |  |  |
|  | **HC > Concussion** | | | **t > 0** | | | **t < 0** | | | **t > 0** | | |  |  |  |
| **JHU white matter** | **Vol (%)** | **t** | **max g** | **Vol (%)** | **t** | **max ρ** | **Vol (%)** | **t** | **max ρ** | **Vol (%)** | **t** | **max ρ** |  |  |  |
| Anterior Thalamic Radiation L | 3.45 | 2.934 | 1.436 | 0.78 | 3.092 | 0.791 | 0.30 | -3.522 | -0.668 | 6.43 | 2.185 | 0.661 |  |  |  |
| Anterior Thalamic Radiation R | 7.20 | 2.627 | 1.148 | 0.33 | 2.760 | 0.644 | — | — | — | 12.52 | 2.372 | 0.738 |  |  |  |
| Cortical spinal tract L | — | — | — | 1.72 | 3.064 | 0.733 | — | — | — | 2.94 | 2.344 | 0.531 |  |  |  |
| Cortical spinal tract R | 0.29 | 2.463 | 0.812 | 1.94 | 3.043 | 0.824 | — | — | — | 5.63 | 2.625 | 0.686 |  |  |  |
| Cingulum cingulate gyrus L | 2.14 | 2.923 | 1.217 | 4.44 | 2.780 | 0.734 | — | — | — | 10.93 | 2.089 | 0.550 |  |  |  |
| Cingulum cingulate gyrus R | — | — | — | 1.07 | 2.607 | 0.664 | — | — | — | 11.20 | 2.124 | 0.446 |  |  |  |
| Cingulum Hippo L | — | — | — | 10.48 | 3.020 | 0.647 | — | — | — | — | — | — |  |  |  |
| Forceps Major | — | — | — | 3.01 | 2.963 | 0.769 | — | — | — | 0.72 | 2.760 | 0.716 |  |  |  |
| Forceps Minor | 4.00 | 2.885 | 1.412 | 1.42 | 2.664 | 0.545 | 0.40 | -3.450 | -0.686 | 20.58 | 2.268 | 0.669 |  |  |  |
| Inferior fronto-occipital fasc L | 2.60 | 2.795 | 1.537 | 4.76 | 2.982 | 0.720 | 0.75 | -3.848 | -0.823 | 8.20 | 2.334 | 0.694 |  |  |  |
| Inferior fronto-occipital fasc R | 2.94 | 2.546 | 1.145 | 0.73 | 2.924 | 0.674 | — | — | — | 8.07 | 2.228 | 0.665 |  |  |  |
| Inferior Longitudinal fasc L | 1.86 | 2.831 | 1.425 | 4.31 | 3.031 | 0.715 | 0.63 | -4.024 | -0.823 | 4.95 | 2.587 | 0.710 |  |  |  |
| Inferior Longitudinal fasc R | 0.71 | 2.819 | 1.282 | — | — | — | — | — | — | 0.20 | 2.106 | 0.510 |  |  |  |
| Superior Longitudinal fasc L | 0.21 | 2.978 | 1.388 | 1.43 | 2.677 | 0.697 | — | — | — | 4.47 | 2.397 | 0.752 |  |  |  |
| Superior Longitudinal fasc R | 0.59 | 2.333 | 0.810 | 3.22 | 3.115 | 0.804 | — | — | — | 5.94 | 2.308 | 0.646 |  |  |  |
| Uncinate fasc L | 4.10 | 2.702 | 1.237 | — | — | — | 0.19 | -3.367 | -0.637 | 7.18 | 2.263 | 0.604 |  |  |  |
| Uncinate fasc R | 3.77 | 2.526 | 0.958 | — | — | — | — | — | — | 9.88 | 2.094 | 0.493 |  |  |  |
| Sup Longitudinal fasc temporal L | 0.36 | 2.964 | 1.334 | 0.97 | 2.754 | 0.678 | 0.13 | -4.363 | -0.820 | 5.67 | 2.390 | 0.749 |  |  |  |
| Sup Longitudinal fasc temporal R | 0.16 | 2.525 | 0.880 | 0.39 | 3.002 | 0.642 | — | — | — | 6.72 | 2.168 | 0.637 |  |  |  |
| **ICBM-DTI 81** | **Vol (%)** | **t** | **max g** | **Vol (%)** | **t** | **max ρ** | **Vol (%)** | **t** | **max ρ** | **Vol (%)** | **t** | **max ρ** |  |  |  |
| Middle cerebellar peduncle | — | — | — | 9.92 | 2.680 | 0.705 | — | — | — | — | — | — |  |  |  |
| Genu of corpus callosum | 2.90 | 2.480 | 0.913 | 5.40 | 2.725 | 0.545 | — | — | — | 18.60 | 2.584 | 0.452 |  |  |  |
| Body of corpus callosum | — | — | — | 12.44 | 3.594 | 0.774 | 0.63 | -3.382 | -0.660 | 11.53 | 2.614 | 0.603 |  |  |  |
| Splenium of corpus callosum | — | — | — | 26.11 | 3.181 | 0.802 | 1.46 | -3.753 | -0.712 | 5.42 | 2.537 | 0.496 |  |  |  |
| Medial lemniscus R | — | — | — | 2.46 | 2.890 | 0.601 | — | — | — | — | — | — |  |  |  |
| Inferior cerebellar peduncle R | — | — | — | 22.21 | 3.272 | 0.777 | — | — | — | — | — | — |  |  |  |
| Superior cerebellar peduncle R | — | — | — | 14.31 | 2.923 | 0.656 | — | — | — | — | — | — |  |  |  |
| Cerebral peduncle L | — | — | — | 6.06 | 3.783 | 0.733 | — | — | — | — | — | — |  |  |  |
| Anterior limb of internal capsule R | 9.53 | 2.687 | 1.042 | — | — | — | — | — | — | 6.18 | 3.283 | 0.727 |  |  |  |
| Anterior limb of internal capsule L | 4.61 | 3.016 | 1.393 | — | — | — | — | — | — | — | — | — |  |  |  |
| Posterior limb of internal capsule R | 1.25 | 2.502 | 0.886 | — | — | — | — | — | — | — | — | — |  |  |  |
| Posterior limb of internal capsule L | — | — | — | 0.48 | 3.007 | 0.550 | — | — | — | — | — | — |  |  |  |
| Retrolenticular part of internal capsule R | 2.31 | 2.476 | 0.880 | — | — | — | — | — | — | — | — | — |  |  |  |
| Retrolenticular part of internal capsule L | — | — | — | 2.03 | 2.566 | 0.589 | — | — | — | 6.12 | 3.068 | 0.463 |  |  |  |
| Anterior corona radiata R | 11.53 | 2.440 | 0.801 | — | — | — | — | — | — | 29.58 | 2.592 | 0.649 |  |  |  |
| Anterior corona radiata L | 6.10 | 2.855 | 1.537 | 0.25 | 2.806 | 0.363 | — | — | — | 20.88 | 2.592 | 0.622 |  |  |  |
| Superior corona radiata R | 10.39 | 2.638 | 0.979 | 5.89 | 3.509 | 0.828 | — | — | — | 16.08 | 2.751 | 0.672 |  |  |  |
| Superior corona radiata L | — | — | — | 6.35 | 2.730 | 0.664 | — | — | — | 2.14 | 2.751 | 0.475 |  |  |  |
| Posterior corona radiata R | 1.85 | 3.129 | 1.325 | 11.78 | 2.932 | 0.664 | — | — | — | 38.25 | 3.071 | 0.686 |  |  |  |
| Posterior corona radiata L | — | — | — | 6.11 | 2.690 | 0.649 | — | — | — | 24.77 | 3.016 | 0.656 |  |  |  |
| Posterior thalamic radiation R | 1.86 | 2.709 | 1.426 | 0.40 | 2.814 | 0.581 | — | — | — | — | — | — |  |  |  |
| Posterior thalamic radiation L | 3.47 | 2.942 | 1.279 | 10.16 | 3.275 | 0.713 | 0.40 | -3.633 | -0.731 | 2.94 | 2.903 | 0.419 |  |  |  |
| Sagittal stratum R | 0.67 | 2.682 | 1.020 | — | — | — | — | — | — | 0.99 | 2.504 | 0.510 |  |  |  |
| Sagittal stratum L | — | — | — | 10.44 | 2.654 | 0.657 | 1.12 | -3.792 | -0.778 | 17.12 | 2.850 | 0.562 |  |  |  |
| External capsule R | 4.49 | 2.260 | 0.751 | — | — | — | — | — | — | 12.60 | 2.458 | 0.445 |  |  |  |
| External capsule L | — | — | — | 1.04 | 2.603 | 0.604 | — | — | — | 5.33 | 3.011 | 0.542 |  |  |  |
| Cingulum (cingulate gyrus) R | — | — | — | 1.62 | 3.317 | 0.749 | — | — | — | 14.65 | 2.633 | 0.393 |  |  |  |
| Cingulum (cingulate gyrus) L | — | — | — | 0.25 | 2.902 | 0.571 | — | — | — | 6.87 | 2.381 | 0.357 |  |  |  |
| Cingulum (hippocampus) L | — | — | — | 15.15 | 2.939 | 0.589 | — | — | — | — | — | — |  |  |  |
| Fornix (cres) / Stria terminalis R | — | — | — | — | — | — | — | — | — | 5.96 | 2.320 | 0.342 |  |  |  |
| Fornix (cres) / Stria terminalis L | — | — | — | 17.24 | 2.805 | 0.647 | — | — | — | 8.00 | 2.876 | 0.396 |  |  |  |
| Superior longitudinal fasciculus R | 1.01 | 2.351 | 0.813 | 0.77 | 2.723 | 0.599 | — | — | — | 10.58 | 2.575 | 0.637 |  |  |  |
| Superior longitudinal fasciculus L | 0.32 | 2.922 | 1.275 | 3.45 | 2.678 | 0.677 | — | — | — | 15.06 | 2.890 | 0.752 |  |  |  |
| Superior fronto-occipital fasciculus R | 14.60 | 2.273 | 0.782 | — | — | — | — | — | — | — | — | — |  |  |  |
| Uncinate fasciculus R | — | — | — | — | — | — | — | — | — | 8.42 | 2.262 | 0.420 |  |  |  |
| Uncinate fasciculus L | 2.13 | 2.560 | 0.992 | — | — | — | — | — | — | — | — | — |  |  |  |
| Tapetum R | — | — | — | 1.34 | 2.825 | 0.566 | — | — | — | — | — | — |  |  |  |

**Supplementary Table 1**

Voxel-wise group comparisons and clinical correlations with MoCA and GOS-E scores for DTI and fw-corrected DTI metrics. The table presents the complete results for fw-FA, fw-F, and conventional FA based on the Johns Hopkins University (JHU) white matter atlas and the ICBM-DTI-81 atlas. For each significant cluster (FDR < 0.05), the percentage of the white matter ROI volume (Vol %), mean t-value, and maximum effect-sizes (max g or max ρ) are reported.

**Sup. Table 2**

| **DKI: KFA** | | | | | | | | | | | | | | | |
| --- | --- | --- | --- | --- | --- | --- | --- | --- | --- | --- | --- | --- | --- | --- | --- |
|  | **KFA** | | | | | | **KFA - MoCA correlation** | | | | | | **KFA - GOS-E correlation** | | |
|  | **HC > Concussion** | | | **HC < Concussion** | | | **t > 0** | | | **t < 0** | | | **t > 0** | | |
| **JHU white matter** | **Vol (%)** | **t** | **max g** | **Vol (%)** | **t** | **max g** | **Vol (%)** | **t** | **max ρ** | **Vol (%)** | **t** | **max ρ** | **Vol (%)** | **t** | **max ρ** |
| Anterior Thalamic Radiation L | 5.68 | 2.566 | 1.035 | — | — | — | 1.41 | 2.566 | 0.791 | 0.20 | -2.954 | -0.660 | 1.35 | 3.039 | 0.716 |
| Anterior Thalamic Radiation R | 4.67 | 2.727 | 0.795 | **—** | **—** | **—** | 0.33 | 2.300 | 0.644 | **—** | **—** | **—** | 2.80 | 2.768 | 0.649 |
| Cortical spinal tract L | 0.92 | 2.212 | 0.627 | **—** | **—** | **—** | 1.72 | 2.553 | 0.733 | **—** | **—** | **—** | **—** | **—** | **—** |
| Cortical spinal tract R | 2.54 | 2.283 | 0.717 | **—** | **—** | **—** | 1.94 | 2.536 | 0.824 | **—** | **—** | **—** | **—** | **—** | **—** |
| Cingulum cingulate gyrus L | 4.34 | 2.571 | 0.640 | **—** | **—** | **—** | 4.44 | 2.316 | 0.734 | **—** | **—** | **—** | 4.46 | 3.732 | 0.841 |
| Cingulum cingulate gyrus R | 0.93 | 2.577 | 0.666 | **—** | **—** | **—** | 1.07 | 2.172 | 0.664 | **—** | **—** | **—** | 10.32 | 2.765 | 0.691 |
| Cingulum Hippo L | 4.83 | 2.935 | 0.591 | **—** | **—** | **—** | 10.48 | 2.516 | 0.647 | **—** | **—** | **—** | 9.09 | 3.822 | 0.807 |
| Cingulum Hippo R | — | — | — | **—** | **—** | **—** | **—** | **—** | **—** | **—** | **—** | **—** | 13.87 | 3.832 | 0.791 |
| Forceps Major | — | — | — | 1.68 | -3.289 | -1.422 | 3.01 | 2.469 | 0.769 | **—** | **—** | **—** | 0.92 | 3.571 | 0.744 |
| Forceps Minor | 3.80 | 2.837 | 0.795 | **—** | **—** | **—** | 1.42 | 2.004 | 0.545 | **—** | **—** | **—** | 6.23 | 3.081 | 0.805 |
| Inferior fronto-occipital fasc L | 1.48 | 2.462 | 0.713 | **—** | **—** | **—** | 5.35 | 2.492 | 0.720 | 0.75 | -2.954 | -0.813 | 3.86 | 3.181 | 0.744 |
| Inferior fronto-occipital fasc R | 0.59 | 2.328 | 0.571 | **—** | **—** | **—** | 1.21 | 2.416 | 0.674 | **—** | **—** | **—** | 1.23 | 2.653 | 0.649 |
| Inferior Longitudinal fasc L | 2.91 | 2.542 | 0.980 | 0.22 | -3.419 | -1.309 | 4.31 | 2.526 | 0.715 | 0.63 | -3.117 | -0.813 | 5.79 | 3.247 | 0.760 |
| Inferior Longitudinal fasc R | 1.51 | 2.483 | 0.722 | **—** | **—** | **—** | **—** | **—** | **—** | **—** | **—** | **—** | **—** | **—** | **—** |
| Superior Longitudinal fasc L | 0.90 | 2.386 | 0.522 | 0.60 | -3.394 | -0.926 | 1.85 | 2.352 | 0.758 | **—** | **—** | **—** | 4.76 | 3.231 | 0.751 |
| Superior Longitudinal fasc R | 1.15 | 2.401 | 0.897 | 0.87 | -3.293 | -1.607 | 3.22 | 2.595 | 0.804 | **—** | **—** | **—** | 0.55 | 3.881 | 0.760 |
| Uncinate fasc L | 4.45 | 2.618 | 0.980 | **—** | **—** | **—** | 0.99 | 2.585 | 0.663 | 0.18 | -2.957 | -0.637 | **—** | **—** | **—** |
| Uncinate fasc R | 1.84 | 2.635 | 0.724 | **—** | **—** | **—** | **—** | **—** | **—** | **—** | **—** | **—** | 0.85 | 2.644 | 0.580 |
| Sup Longitudinal fasc temporal L | 2.11 | 2.390 | 0.522 | **—** | **—** | **—** | 1.93 | 2.532 | 0.758 | 0.13 | -3.181 | -0.810 | 4.92 | 3.169 | 0.751 |
| Sup Longitudinal fasc temporal R | 2.07 | 2.383 | 0.525 | 0.37 | -3.293 | -1.088 | 0.39 | 2.502 | 0.642 | **—** | **—** | **—** | 0.37 | 3.500 | 0.731 |
| **ICBM-DTI 81** | **Vol (%)** | **t** | **max g** | **Vol (%)** | **t** | **max g** | **Vol (%)** | **t** | **max ρ** | **Vol (%)** | **t** | **max ρ** | **Vol (%)** | **t** | **max ρ** |
| Middle cerebellar peduncle | 12.52 | 2.356 | 0.756 | **—** | **—** | **—** | 9.92 | 2.234 | 0.705 | **—** | **—** | **—** | **—** | **—** | **—** |
| Pontine crossing tract | 0.40 | 2.142 | 0.546 | **—** | **—** | **—** | **—** | **—** | **—** | **—** | **—** | **—** | **—** | **—** | **—** |
| Genu of corpus callosum | 0.80 | 2.623 | 0.469 | **—** | **—** | **—** | 5.40 | 2.021 | 0.545 | **—** | **—** | **—** | 10.61 | 3.237 | 0.614 |
| Body of corpus callosum | 0.34 | 2.323 | 0.486 | **—** | **—** | **—** | 12.44 | 2.161 | 0.674 | 0.63 | -2.943 | -0.660 | 5.57 | 2.958 | 0.564 |
| Splenium of corpus callosum | 0.20 | 2.757 | 0.736 | 0.61 | -3.299 | -1.422 | 26.11 | 2.651 | 0.802 | 1.46 | -2.942 | -0.712 | **—** | **—** | **—** |
| Corticospinal tract R | 3.82 | 2.177 | 0.497 | **—** | **—** | **—** | **—** | **—** | **—** | **—** | **—** | **—** | **—** | **—** | **—** |
| Corticospinal tract L | 0.44 | 2.068 | 0.513 | **—** | **—** | **—** | **—** | **—** | **—** | **—** | **—** | **—** | **—** | **—** | **—** |
| Medial lemniscus R | 1.01 | 2.028 | 0.482 | **—** | **—** | **—** | 2.46 | 2.158 | 0.601 | **—** | **—** | **—** | **—** | **—** | **—** |
| Inferior cerebellar peduncle R | 0.62 | 2.144 | 0.491 | **—** | **—** | **—** | 22.21 | 2.727 | 0.777 | **—** | **—** | **—** | **—** | **—** | **—** |
| Superior cerebellar peduncle R | — | — | — | **—** | **—** | **—** | 14.31 | 2.435 | 0.656 | **—** | **—** | **—** | **—** | **—** | **—** |
| Cerebral peduncle R | 1.67 | 2.481 | 0.656 | **—** | **—** | **—** | **—** | **—** | **—** | **—** | **—** | **—** | **—** | **—** | **—** |
| Cerebral peduncle L | — | — | — | **—** | **—** | **—** | 6.06 | 3.153 | 0.733 | **—** | **—** | **—** | **—** | **—** | **—** |
| Anterior limb of internal capsule R | 4.84 | 2.410 | 0.495 | **—** | **—** | **—** | **—** | **—** | **—** | **—** | **—** | **—** | **—** | **—** | **—** |
| Anterior limb of internal capsule L | 13.42 | 2.452 | 0.503 | **—** | **—** | **—** | **—** | **—** | **—** | **—** | **—** | **—** | **—** | **—** | **—** |
| Posterior limb of internal capsule R | 2.69 | 2.313 | 0.506 | **—** | **—** | **—** | **—** | **—** | **—** | **—** | **—** | **—** | **—** | **—** | **—** |
| Posterior limb of internal capsule L | — | — | — | **—** | **—** | **—** | 0.48 | 2.506 | 0.550 | **—** | **—** | **—** | **—** | **—** | **—** |
| Retrolenticular part of internal capsule R | — | — | — | **—** | **—** | **—** | 0.68 | 2.361 | 0.614 | **—** | **—** | **—** | **—** | **—** | **—** |
| Retrolenticular part of internal capsule L | 7.41 | 2.471 | 0.965 | **—** | **—** | **—** | 2.03 | 2.138 | 0.589 | **—** | **—** | **—** | 4.21 | 3.268 | 0.544 |
| Anterior corona radiata R | 11.39 | 2.677 | 1.072 | **—** | **—** | **—** | **—** | **—** | **—** | **—** | **—** | **—** | 11.67 | 3.183 | 0.669 |
| Anterior corona radiata L | 6.12 | 2.420 | 0.586 | **—** | **—** | **—** | 2.92 | 2.496 | 0.663 | **—** | **—** | **—** | 1.04 | 3.585 | 0.662 |
| Superior corona radiata R | 5.64 | 2.501 | 0.839 | **—** | **—** | **—** | 5.89 | 2.924 | 0.828 | **—** | **—** | **—** | 11.72 | 3.493 | 0.721 |
| Superior corona radiata L | 0.79 | 2.299 | 0.498 | **—** | **—** | **—** | 6.35 | 2.275 | 0.664 | **—** | **—** | **—** | **—** | **—** | **—** |
| Posterior corona radiata R | 2.68 | 3.469 | 0.875 | 0.21 | -3.292 | -1.052 | 11.78 | 2.444 | 0.664 | **—** | **—** | **—** | **—** | **—** | **—** |
| Posterior corona radiata L | — | — | — | **—** | **—** | **—** | 6.11 | 2.242 | 0.649 | **—** | **—** | **—** | 14.40 | 3.413 | 0.765 |
| Posterior thalamic radiation R | 0.35 | 2.079 | 0.482 | 0.23 | -3.301 | -1.002 | 0.40 | 2.011 | 0.581 | **—** | **—** | **—** | **—** | **—** | **—** |
| Posterior thalamic radiation L | 2.54 | 2.362 | 0.560 | **—** | **—** | **—** | 10.16 | 2.729 | 0.713 | 0.40 | -3.117 | -0.731 | 9.38 | 3.289 | 0.738 |
| Sagittal stratum R | 0.36 | 2.096 | 0.543 | **—** | **—** | **—** | 0.13 | 2.368 | 0.604 | **—** | **—** | **—** | **—** | **—** | **—** |
| Sagittal stratum L | 0.18 | 2.570 | 0.498 | **—** | **—** | **—** | 10.44 | 2.212 | 0.657 | 1.12 | -3.117 | -0.778 | 27.25 | 3.541 | 0.706 |
| External capsule R | 4.83 | 2.384 | 0.631 | **—** | **—** | **—** | 1.96 | 2.393 | 0.629 | **—** | **—** | **—** | **—** | **—** | **—** |
| External capsule L | 0.16 | 2.287 | 0.471 | **—** | **—** | **—** | 1.04 | 2.169 | 0.604 | **—** | **—** | **—** | **—** | **—** | **—** |
| Cingulum (cingulate gyrus) R | — | — | — | **—** | **—** | **—** | 1.62 | 2.764 | 0.749 | **—** | **—** | **—** | 10.67 | 2.971 | 0.628 |
| Cingulum (cingulate gyrus) L | 2.76 | 2.552 | 0.492 | **—** | **—** | **—** | 0.25 | 2.001 | 0.571 | **—** | **—** | **—** | **—** | **—** | **—** |
| Cingulum (hippocampus) R | — | — | — | **—** | **—** | **—** | **—** | **—** | **—** | **—** | **—** | **—** | 22.17 | 4.256 | 0.791 |
| Cingulum (hippocampus) L | — | — | — | **—** | **—** | **—** | 15.15 | 2.449 | 0.589 | **—** | **—** | **—** | 15.32 | 4.200 | 0.807 |
| Fornix (cres) / Stria terminalis R | — | — | — | **—** | **—** | **—** | **—** | **—** | **—** | **—** | **—** | **—** | 20.91 | 3.942 | 0.791 |
| Fornix (cres) / Stria terminalis L | 3.02 | 2.502 | 0.471 | **—** | **—** | **—** | 17.24 | 2.337 | 0.647 | **—** | **—** | **—** | 17.51 | 3.812 | 0.746 |
| Superior longitudinal fasciculus R | 0.94 | 2.334 | 0.809 | 0.80 | -3.293 | -1.145 | 0.77 | 2.102 | 0.599 | **—** | **—** | **—** | 2.07 | 4.182 | 0.760 |
| Superior longitudinal fasciculus L | 2.42 | 2.278 | 0.500 | **—** | **—** | **—** | 3.53 | 2.238 | 0.677 | **—** | **—** | **—** | 9.17 | 3.345 | 0.682 |
| Superior fronto-occipital fasciculus L | 28.99 | 2.489 | 0.647 | **—** | **—** | **—** | **—** | **—** | **—** | **—** | **—** | **—** | **—** | **—** | **—** |
| Uncinate fasciculus L | 24.20 | 2.388 | 0.670 | **—** | **—** | **—** | **—** | **—** | **—** | **—** | **—** | **—** | **—** | **—** | **—** |
| Tapetum R | — | — | — | **—** | **—** | **—** | 1.34 | 1.990 | 0.566 | **—** | **—** | **—** | **—** | **—** | **—** |
| Tapetum L | — | — | — | **—** | **—** | **—** | 0.17 | 2.124 | 0.544 | **—** | **—** | **—** | 0.83 | 2.800 | 0.423 |
| **DKI: MK** | | | | | | | | | | | | | | | |
|  | **MK** | | | | | | **MK - MoCA correlation** | | | | | | **MK - GOS-E correlation** | | |
|  | **HC > Concussion** | | | **HC < Concussion** | | | **t > 0** | | | **t < 0** | | | **t > 0** | | |
| **JHU white matter** | **Vol (%)** | **t** | **max g** | **Vol (%)** | **t** | **max g** | **Vol (%)** | **t** | **max ρ** | **Vol (%)** | **t** | **max ρ** | **Vol (%)** | **t** | **max ρ** |
| Anterior Thalamic Radiation L | — | — | — | 0.26 | -3.357 | -1.015 | 1.94 | 3.713 | 0.606 | — | — | — | 1.44 | 3.486 | 0.842 |
| Anterior Thalamic Radiation R | — | — | — | — | — | — | 2.08 | 3.797 | 0.607 | — | — | — | — | — | — |
| Cortical spinal tract L | 0.37 | 2.553 | 0.553 | — | — | — | — | — | — | — | — | — | — | — | — |
| Cortical spinal tract R | — | — | — | — | — | — | — | — | — | — | — | — | 0.34 | 4.251 | 0.756 |
| Cingulum cingulate gyrus L | — | — | — | — | — | — | 11.52 | 4.105 | 0.655 | — | — | — | — | — | — |
| Cingulum cingulate gyrus R | — | — | — | — | — | — | 6.75 | 3.647 | 0.718 | — | — | — | — | — | — |
| Forceps Major | 0.41 | 2.600 | 0.856 | — | — | — | 0.75 | 3.972 | 0.714 | — | — | — | — | — | — |
| Forceps Minor | 0.32 | 2.503 | 0.834 | — | — | — | 7.21 | 4.148 | 0.695 | — | — | — | 0.21 | 3.419 | 0.444 |
| Inferior fronto-occipital fasc L | 1.05 | 2.485 | 1.295 | — | — | — | 0.37 | 3.713 | 0.604 | 0.47 | -3.885 | -0.721 | 0.27 | 3.525 | 0.773 |
| Inferior fronto-occipital fasc R | 0.85 | 2.557 | 0.956 | — | — | — | 0.69 | 3.597 | 0.669 | 0.45 | -3.975 | -0.737 | — | — | — |
| Inferior Longitudinal fasc L | 1.04 | 2.502 | 1.295 | — | — | — | — | — | — | — | — | — | — | — | — |
| Inferior Longitudinal fasc R | 1.28 | 2.451 | 0.819 | — | — | — | — | — | — | — | — | — | — | — | — |
| Superior Longitudinal fasc R | 0.27 | 2.853 | 0.759 | — | — | — | — | — | — | — | — | — | 0.14 | 4.016 | 0.622 |
| Uncinate fasc L | 0.79 | 2.508 | 0.781 | — | — | — | — | — | — | 0.72 | -3.793 | -0.693 | 0.45 | 3.433 | 0.751 |
| Uncinate fasc R | 0.23 | 2.529 | 0.956 | — | — | — | 1.85 | 4.170 | 0.573 | — | — | — | — | — | — |
| **ICBM-DTI 81** | **Vol (%)** | **t** | **max g** | **Vol (%)** | **t** | **max g** | **Vol (%)** | **t** | **max ρ** | **Vol (%)** | **t** | **max ρ** | **Vol (%)** | **t** | **max ρ** |
| Genu of corpus callosum | — | — | — | — | — | — | 11.41 | 4.148 | 0.695 | — | — | — | — | — | — |
| Body of corpus callosum | — | — | — | — | — | — | 1.40 | 2.774 | 0.580 | — | — | — | 0.61 | 3.687 | 0.506 |
| Splenium of corpus callosum | — | — | — | — | — | — | 13.22 | 4.105 | 0.775 | — | — | — | — | — | — |
| Cerebral peduncle L | 2.94 | 2.553 | 0.553 | — | — | — | — | — | — | — | — | — | 2.99 | 3.517 | 0.783 |
| Posterior limb of internal capsule R | — | — | — | — | — | — | 0.24 | 2.817 | 0.485 | — | — | — | — | — | — |
| Posterior limb of internal capsule L | — | — | — | — | — | — | — | — | — | — | — | — | 7.12 | 3.916 | 0.842 |
| Anterior corona radiata R | — | — | — | — | — | — | 8.21 | 4.118 | 0.693 | — | — | — | — | — | — |
| Anterior corona radiata L | — | — | — | — | — | — | 12.43 | 3.431 | 0.620 | — | — | — | 0.99 | 3.568 | 0.773 |
| Posterior corona radiata R | — | — | — | — | — | — | 1.23 | 3.142 | 0.657 | — | — | — | — | — | — |
| Posterior thalamic radiation R | — | — | — | — | — | — | 0.63 | 2.889 | 0.653 | — | — | — | — | — | — |
| Posterior thalamic radiation L | 0.25 | 2.563 | 0.906 | — | — | — | — | — | — | — | — | — | — | — | — |
| Sagittal stratum R | 4.08 | 2.516 | 0.816 | — | — | — | — | — | — | — | — | — | — | — | — |
| External capsule L | — | — | — | — | — | — | — | — | — | — | — | — | 0.34 | 3.389 | 0.659 |
| Cingulum (cingulate gyrus) R | — | — | — | — | — | — | 4.74 | 3.232 | 0.675 | — | — | — | — | — | — |
| Cingulum (cingulate gyrus) L | — | — | — | — | — | — | 0.87 | 2.802 | 0.569 | — | — | — | — | — | — |
| Fornix (cres) / Stria terminalis L | — | — | — | 0.36 | -3.299 | -0.599 | — | — | — | — | — | — | — | — | — |
| **DKI: MKT** | | | | | | | | | | | | | | | |
|  | **MKT** | | | | | | **MKT - MoCA correlation** | | | | | | **MKT - GOS-E correlation** | | |
|  | **HC > Concussion** | | | **HC < Concussion** | | | **t > 0** | | | **t < 0** | | | **t > 0** | | |
| **JHU white matter** | **Vol (%)** | **t** | **max g** | **Vol (%)** | **t** | **max g** | **Vol (%)** | **t** | **max ρ** | **Vol (%)** | **t** | **max ρ** | **Vol (%)** | **t** | **max ρ** |
| Anterior Thalamic Radiation L | 0.40 | 2.311 | 0.855 | 0.20 | -3.177 | -0.982 | 2.20 | 3.912 | 0.633 | — | — | — | — | — | — |
| Anterior Thalamic Radiation R | — | — | — | — | — | — | 2.58 | 3.891 | 0.601 | — | — | — | 0.52 | 3.706 | 0.792 |
| Cortical spinal tract L | — | — | — | — | — | — | — | — | — | — | — | — | 0.36 | 3.584 | 0.614 |
| Cingulum cingulate gyrus L | — | — | — | — | — | — | 15.86 | 3.894 | 0.654 | — | — | — | — | — | — |
| Cingulum cingulate gyrus R | — | — | — | — | — | — | 7.71 | 3.859 | 0.732 | — | — | — | — | — | — |
| Cingulum Hippo L | 2.01 | 3.046 | 0.944 | — | — | — | — | — | — | — | — | — | — | — | — |
| Cingulum Hippo R | 2.75 | 2.258 | 0.919 | — | — | — | — | — | — | — | — | — | — | — | — |
| Forceps Major | 1.03 | 3.312 | 1.090 | 0.47 | -2.940 | -1.271 | — | — | — | — | — | — | — | — | — |
| Forceps Minor | 0.27 | 2.446 | 0.752 | — | — | — | 10.01 | 3.865 | 0.673 | 0.21 | -3.103 | -0.537 | — | — | — |
| Inferior fronto-occipital fasc L | 2.68 | 3.103 | 1.313 | — | — | — | 0.40 | 3.882 | 0.628 | 1.23 | -2.978 | -0.750 | — | — | — |
| Inferior fronto-occipital fasc R | 2.14 | 3.462 | 1.139 | — | — | — | 0.30 | 3.701 | 0.508 | 0.53 | -3.097 | -0.756 | — | — | — |
| Inferior Longitudinal fasc L | 3.43 | 3.275 | 1.313 | — | — | — | — | — | — | — | — | — | — | — | — |
| Inferior Longitudinal fasc R | 4.28 | 3.022 | 1.139 | — | — | — | — | — | — | — | — | — | — | — | — |
| Superior Longitudinal fasc L | 0.55 | 3.185 | 0.994 | — | — | — | — | — | — | — | — | — | — | — | — |
| Superior Longitudinal fasc R | 1.15 | 3.438 | 0.939 | — | — | — | — | — | — | — | — | — | — | — | — |
| Uncinate fasc L | 1.04 | 2.446 | 0.725 | — | — | — | 0.13 | 3.964 | 0.590 | 1.53 | -2.978 | -0.750 | — | — | — |
| Uncinate fasc R | — | — | — | — | — | — | 0.37 | 3.566 | 0.444 | — | — | — | — | — | — |
| Sup Longitudinal fasc temporal L | 0.70 | 2.749 | 0.994 | — | — | — | — | — | — | — | — | — | — | — | — |
| Sup Longitudinal fasc temporal R | 0.10 | 2.346 | 0.484 | — | — | — | — | — | — | — | — | — | — | — | — |
| **ICBM-DTI 81** | **Vol (%)** | **t** | **max g** | **Vol (%)** | **t** | **max g** | **Vol (%)** | **t** | **max ρ** | **Vol (%)** | **t** | **max ρ** | **Vol (%)** | **t** | **max ρ** |
| Genu of corpus callosum | — | — | — | — | — | — | 22.18 | 3.770 | 0.673 | — | — | — | — | — | — |
| Body of corpus callosum | — | — | — | — | — | — | 14.10 | 3.644 | 0.601 | — | — | — | — | — | — |
| Splenium of corpus callosum | — | — | — | 2.16 | -2.936 | -1.271 | 2.52 | 4.789 | 0.698 | — | — | — | — | — | — |
| Corticospinal tract L | — | — | — | — | — | — | — | — | — | — | — | — | 1.68 | 3.616 | 0.575 |
| Cerebral peduncle L | — | — | — | — | — | — | — | — | — | — | — | — | 1.23 | 3.473 | 0.614 |
| Anterior limb of internal capsule R | — | — | — | — | — | — | 4.40 | 3.847 | 0.436 | — | — | — | 0.13 | 3.354 | 0.714 |
| Posterior limb of internal capsule R | — | — | — | — | — | — | — | — | — | — | — | — | 3.06 | 3.742 | 0.792 |
| Posterior limb of internal capsule L | — | — | — | — | — | — | — | — | — | — | — | — | 0.19 | 3.718 | 0.587 |
| Retrolenticular part of internal capsule L | — | — | — | — | — | — | — | — | — | — | — | — | 1.26 | 3.828 | 0.600 |
| Anterior corona radiata R | — | — | — | — | — | — | 12.79 | 3.969 | 0.657 | — | — | — | — | — | — |
| Anterior corona radiata L | — | — | — | — | — | — | 15.19 | 3.728 | 0.633 | — | — | — | — | — | — |
| Posterior thalamic radiation R | 1.81 | 2.362 | 0.579 | — | — | — | — | — | — | — | — | — | — | — | — |
| Posterior thalamic radiation L | 0.73 | 2.519 | 0.715 | — | — | — | — | — | — | — | — | — | — | — | — |
| Sagittal stratum R | 2.60 | 2.457 | 0.571 | — | — | — | — | — | — | — | — | — | — | — | — |
| Sagittal stratum L | 1.52 | 2.502 | 0.736 | — | — | — | — | — | — | — | — | — | — | — | — |
| Cingulum (cingulate gyrus) R | — | — | — | — | — | — | 5.17 | 3.762 | 0.636 | — | — | — | — | — | — |
| Cingulum (cingulate gyrus) L | — | — | — | — | — | — | 4.33 | 3.432 | 0.580 | — | — | — | — | — | — |
| Cingulum (hippocampus) R | 5.58 | 2.258 | 0.919 | — | — | — | — | — | — | — | — | — | — | — | — |
| Cingulum (hippocampus) L | 1.30 | 2.242 | 0.770 | — | — | — | — | — | — | — | — | — | — | — | — |
| Fornix (cres) / Stria terminalis L | — | — | — | 0.18 | -3.192 | -0.567 | — | — | — | — | — | — | — | — | — |

**Supplementary Table 2**

Voxel-wise group comparisons and clinical correlations with MoCA and GOS-E scores for DKI metrics. The table presents the complete results for KFA, MK, and MKT based on the Johns Hopkins University (JHU) white matter atlas and the ICBM-DTI-81 atlas. For each significant cluster (FDR < 0.05), the percentage of the white matter ROI volume (Vol %), mean t-value, and maximum effect-sizes (max g or max ρ) are reported.

**Sup. Table 3**

| **NODDI: FWF** | | | | | | | | | | | | | | | | | | |
| --- | --- | --- | --- | --- | --- | --- | --- | --- | --- | --- | --- | --- | --- | --- | --- | --- | --- | --- |
|  | **FWF** | | | | | | **FWF - MoCA correlation** | | | | | | **FWF - GOS-E correlation** | | | | | |
|  | HC > Concussion | | | HC < Concussion | | | t > 0 | | | t < 0 | | | t > 0 | | | t < 0 | | |
| **JHU white matter** | Vol (%) | t | max g | Vol (%) | t | max g | Vol (%) | t | max ρ | Vol (%) | t | max ρ | Vol (%) | t | max ρ | Vol (%) | t | max ρ |
| Anterior Thalamic Radiation L | — | — | — | 0.93 | -3.335 | -0.639 | 0.74 | 2.713 | 0.697 | 2.50 | -5.320 | -0.699 | 0.34 | 2.965 | 0.669 | 0.52 | -3.236 | -0.693 |
| Anterior Thalamic Radiation R | — | — | — | 1.88 | -3.012 | -0.913 | 0.84 | 2.737 | 0.620 | 4.97 | -5.312 | -0.707 | — | — | — | 0.83 | -3.896 | -0.647 |
| Cortical spinal tract L | — | — | — | 4.70 | -3.199 | -0.534 | — | — | — | 10.06 | -4.423 | -0.888 | — | — | — | 1.73 | -3.501 | -0.686 |
| Cortical spinal tract R | — | — | — | 2.89 | -2.830 | -0.661 | — | — | — | 13.55 | -4.875 | -0.695 | — | — | — | 2.75 | -2.780 | -0.647 |
| Cingulum cingulate gyrus L | — | — | — | 3.17 | -3.255 | -0.949 | — | — | — | 0.40 | -3.697 | -0.672 | — | — | — | — | — | — |
| Cingulum Hippo R | — | — | — | — | — | — | — | — | — | — | — | — | — | — | — | 1.64 | -3.524 | -0.605 |
| Forceps Major | — | — | — | 0.35 | -3.191 | -0.531 | 1.14 | 2.978 | 0.740 | 3.48 | -4.297 | -0.764 | — | — | — | 2.89 | -4.269 | -0.740 |
| Forceps Minor | — | — | — | 1.60 | -3.093 | -1.000 | 0.73 | 2.585 | 0.635 | — | — | — | — | — | — | 1.93 | -3.649 | -0.670 |
| Inferior fronto-occipital fasc L | — | — | — | 0.52 | -3.313 | -0.596 | — | — | — | 1.57 | -4.789 | -0.784 | — | — | — | 0.37 | -4.408 | -0.566 |
| Inferior fronto-occipital fasc R | 0.58 | 3.906 | 1.197 | 2.52 | -3.375 | -1.052 | 0.26 | 2.557 | 0.617 | — | — | — | — | — | — | 1.00 | -2.699 | -0.551 |
| Inferior Longitudinal fasc L | — | — | — | 0.16 | -3.040 | -0.563 | — | — | — | 2.69 | -4.789 | -0.784 | — | — | — | 0.25 | -4.030 | -0.543 |
| Inferior Longitudinal fasc R | 0.42 | 3.932 | 1.197 | 1.04 | -3.518 | -1.008 | — | — | — | — | — | — | — | — | — | 0.30 | -3.281 | -0.541 |
| Superior Longitudinal fasc L | — | — | — | 1.80 | -3.183 | -0.800 | — | — | — | 2.15 | -5.015 | -0.721 | — | — | — | 0.55 | -2.334 | -0.581 |
| Superior Longitudinal fasc R | — | — | — | 0.36 | -3.551 | -1.040 | — | — | — | 2.89 | -3.666 | -0.697 | — | — | — | 5.61 | -3.787 | -0.772 |
| Uncinate fasc L | — | — | — | 0.33 | -2.961 | -0.613 | — | — | — | 1.34 | -3.102 | -0.665 | — | — | — | — | — | — |
| Uncinate fasc R | — | — | — | 0.81 | -2.786 | -0.598 | — | — | — | — | — | — | — | — | — | — | — | — |
| Sup Longitudinal fasc temporal L | — | — | — | 3.44 | -3.213 | -0.800 | — | — | — | 3.99 | -5.015 | -0.721 | — | — | — | 0.96 | -2.334 | -0.540 |
| Sup Longitudinal fasc temporal R | — | — | — | 1.26 | -3.551 | -1.040 | — | — | — | 1.73 | -3.049 | -0.561 | — | — | — | 7.02 | -4.712 | -0.762 |
| **ICBM-DTI 81** | Vol (%) | t | max g | Vol (%) | t | max g | Vol (%) | t | max ρ | Vol (%) | t | max ρ | Vol (%) | t | max ρ | Vol (%) | t | max ρ |
| Middle cerebellar peduncle | — | — | — | 2.60 | -2.869 | -0.651 | — | — | — | 11.28 | -4.791 | -0.769 | — | — | — | 2.28 | -2.877 | -0.471 |
| Pontine crossing tract | — | — | — | 2.07 | -2.754 | -0.565 | — | — | — | 57.60 | -4.076 | -0.875 | — | — | — | — | — | — |
| Genu of corpus callosum | — | — | — | — | — | — | 0.24 | 2.334 | 0.556 | — | — | — | — | — | — | 10.44 | -3.649 | -0.670 |
| Body of corpus callosum | — | — | — | 0.85 | -3.241 | -0.570 | — | — | — | 0.28 | -3.025 | -0.567 | — | — | — | 2.15 | -4.339 | -0.696 |
| Splenium of corpus callosum | — | — | — | 0.31 | -3.242 | -0.873 | 1.25 | 2.824 | 0.740 | 5.22 | -3.965 | -0.830 | — | — | — | 1.97 | -3.713 | -0.740 |
| Corticospinal tract R | — | — | — | 14.98 | -2.792 | -0.591 | — | — | — | 19.09 | -4.508 | -0.538 | — | — | — | 1.54 | -2.537 | -0.577 |
| Corticospinal tract L | — | — | — | — | — | — | — | — | — | 29.93 | -4.096 | -0.887 | — | — | — | — | — | — |
| Medial lemniscus R | — | — | — | — | — | — | — | — | — | 32.75 | -3.294 | -0.618 | — | — | — | — | — | — |
| Medial lemniscus L | — | — | — | — | — | — | — | — | — | 40.06 | -3.799 | -0.697 | — | — | — | — | — | — |
| Inferior cerebellar peduncle R | — | — | — | 0.72 | -2.652 | -0.570 | — | — | — | 14.57 | -3.058 | -0.645 | — | — | — | — | — | — |
| Inferior cerebellar peduncle L | — | — | — | — | — | — | — | — | — | 17.25 | -4.841 | -0.811 | — | — | — | — | — | — |
| Superior cerebellar peduncle R | — | — | — | — | — | — | — | — | — | 16.03 | -4.875 | -0.514 | — | — | — | — | — | — |
| Superior cerebellar peduncle L | — | — | — | — | — | — | — | — | — | 4.23 | -2.217 | -0.483 | — | — | — | — | — | — |
| Cerebral peduncle R | — | — | — | 2.02 | -2.988 | -0.606 | — | — | — | 15.80 | -4.100 | -0.705 | — | — | — | 7.42 | -2.780 | -0.594 |
| Cerebral peduncle L | — | — | — | — | — | — | — | — | — | 19.53 | -3.472 | -0.561 | — | — | — | 7.11 | -3.372 | -0.645 |
| Anterior limb of internal capsule R | — | — | — | 3.44 | -2.911 | -0.582 | 2.29 | 2.877 | 0.573 | 0.22 | -2.943 | -0.519 | — | — | — | — | — | — |
| Anterior limb of internal capsule L | — | — | — | 4.57 | -3.238 | -0.552 | 4.31 | 2.662 | 0.687 | — | — | — | — | — | — | — | — | — |
| Posterior limb of internal capsule R | — | — | — | 3.01 | -2.866 | -0.661 | 1.23 | 2.671 | 0.561 | — | — | — | — | — | — | — | — | — |
| Posterior limb of internal capsule L | — | — | — | 2.16 | -3.885 | -0.578 | 0.16 | 2.824 | 0.584 | 0.85 | -2.813 | -0.418 | 0.11 | 3.266 | 0.457 | — | — | — |
| Retrolenticular part of internal capsule R | — | — | — | 8.75 | -3.645 | -1.052 | — | — | — | — | — | — | — | — | — | — | — | — |
| Anterior corona radiata R | — | — | — | 12.73 | -3.231 | -0.698 | 3.99 | 2.606 | 0.635 | 1.99 | -2.685 | -0.570 | — | — | — | 0.76 | -3.896 | -0.614 |
| Anterior corona radiata L | — | — | — | 3.96 | -2.990 | -0.538 | — | — | — | — | — | — | — | — | — | — | — | — |
| Superior corona radiata R | — | — | — | — | — | — | — | — | — | 19.47 | -3.808 | -0.587 | — | — | — | 15.23 | -3.170 | -0.706 |
| Superior corona radiata L | — | — | — | 15.78 | -3.539 | -0.534 | — | — | — | 10.78 | -3.377 | -0.689 | — | — | — | — | — | — |
| Posterior corona radiata R | — | — | — | — | — | — | — | — | — | 7.54 | -3.674 | -0.670 | — | — | — | 10.92 | -2.694 | -0.594 |
| Posterior corona radiata L | — | — | — | 0.83 | -3.011 | -0.704 | — | — | — | 0.62 | -3.467 | -0.589 | — | — | — | 3.53 | -2.799 | -0.693 |
| Posterior thalamic radiation R | 3.47 | 3.946 | 1.197 | 1.01 | -3.058 | -0.582 | 0.20 | 2.355 | 0.496 | — | — | — | — | — | — | 4.10 | -2.517 | -0.541 |
| Posterior thalamic radiation L | — | — | — | — | — | — | 1.23 | 3.228 | 0.714 | 4.15 | -2.945 | -0.613 | — | — | — | — | — | — |
| Sagittal stratum R | — | — | — | 1.97 | -3.083 | -0.529 | — | — | — | — | — | — | — | — | — | — | — | — |
| Sagittal stratum L | — | — | — | 0.81 | -3.327 | -0.561 | — | — | — | 4.35 | -4.952 | -0.778 | — | — | — | — | — | — |
| External capsule R | — | — | — | 0.18 | -3.050 | -0.529 | — | — | — | 0.21 | -3.084 | -0.555 | — | — | — | — | — | — |
| External capsule L | — | — | — | 0.79 | -3.596 | -0.574 | — | — | — | — | — | — | — | — | — | — | — | — |
| Cingulum (cingulate gyrus) L | — | — | — | 0.76 | -3.765 | -0.522 | — | — | — | 0.98 | -3.697 | -0.672 | — | — | — | — | — | — |
| Fornix (cres) / Stria terminalis R | — | — | — | — | — | — | — | — | — | 29.36 | -4.008 | -0.686 | — | — | — | — | — | — |
| Fornix (cres) / Stria terminalis L | — | — | — | 1.60 | -3.006 | -0.536 | — | — | — | 8.80 | -2.957 | -0.626 | — | — | — | 22.84 | -3.236 | -0.552 |
| Superior longitudinal fasciculus R | — | — | — | — | — | — | — | — | — | 8.08 | -3.649 | -0.697 | — | — | — | 7.86 | -5.416 | -0.737 |
| Superior longitudinal fasciculus L | — | — | — | 7.42 | -3.113 | -0.641 | — | — | — | 3.32 | -3.427 | -0.570 | — | — | — | 1.88 | -2.331 | -0.502 |
| Superior fronto-occipital fasciculus R | — | — | — | — | — | — | 1.18 | 2.685 | 0.561 | 6.90 | -3.220 | -0.565 | — | — | — | 8.48 | -3.202 | -0.352 |
| Superior fronto-occipital fasciculus L | — | — | — | 13.02 | -3.079 | -0.571 | — | — | — | 0.99 | -2.955 | -0.380 | — | — | — | — | — | — |
| Tapetum R | 0.34 | 3.472 | 0.823 | — | — | — | 23.66 | 2.605 | 0.623 | — | — | — | — | — | — | 24.83 | -3.487 | -0.540 |
| Tapetum L | — | — | — | — | — | — | 4.67 | 2.782 | 0.632 | — | — | — | — | — | — | — | — | — |
| **NODDI: ODI** | | | | | | | | | | | | | | | | | | |
|  | **ODI** | | | | | | **ODI - MoCA correlation** | | | | | | **ODI - GOS-E correlation** | | | | | |
|  | HC > Concussion | | | HC < Concussion | | | t > 0 | | | t < 0 | | | t > 0 | | | t < 0 | | |
| **JHU white matter** | Vol (%) | t | max g | Vol (%) | t | max g | Vol (%) | t | max ρ | Vol (%) | t | max ρ | Vol (%) | t | max ρ | Vol (%) | t | max ρ |
| Anterior Thalamic Radiation L | — | — | — | 2.38 | -2.796 | -1.462 | — | — | — | 1.73 | -3.465 | -0.807 | — | — | — | — | — | — |
| Anterior Thalamic Radiation R | — | — | — | 8.48 | -2.709 | -1.617 | 3.29 | 3.404 | 0.658 | — | — | — | — | — | — | 0.39 | -3.372 | -0.600 |
| Cortical spinal tract R | — | — | — | 2.27 | -2.493 | -0.991 | — | — | — | 1.14 | -3.514 | -0.773 | 0.35 | 3.273 | 0.697 | 1.54 | -3.627 | -0.746 |
| Cingulum cingulate gyrus L | — | — | — | 1.95 | -2.948 | -1.388 | — | — | — | 1.62 | -3.779 | -0.763 | — | — | — | — | — | — |
| Cingulum cingulate gyrus R | — | — | — | — | — | — | — | — | — | 0.31 | -3.240 | -0.659 | — | — | — | — | — | — |
| Forceps Major | 0.11 | 2.987 | 1.018 | — | — | — | — | — | — | 2.86 | -3.412 | -0.753 | — | — | — | — | — | — |
| Forceps Minor | — | — | — | 2.62 | -2.698 | -1.617 | — | — | — | 1.46 | -3.180 | -0.733 | — | — | — | 0.66 | -3.678 | -0.756 |
| Inferior fronto-occipital fasc L | 0.58 | 3.296 | 0.912 | 1.69 | -2.661 | -1.462 | — | — | — | 5.67 | -3.505 | -0.826 | — | — | — | — | — | — |
| Inferior fronto-occipital fasc R | 0.46 | 3.087 | 1.118 | 2.21 | -2.603 | -1.617 | — | — | — | 0.76 | -3.269 | -0.645 | — | — | — | 0.36 | -3.390 | -0.620 |
| Inferior Longitudinal fasc L | 1.04 | 3.317 | 0.912 | 1.58 | -2.784 | -1.177 | — | — | — | 4.38 | -3.461 | -0.773 | — | — | — | — | — | — |
| Inferior Longitudinal fasc R | — | — | — | 0.51 | -3.098 | -0.657 | — | — | — | 0.17 | -3.520 | -0.641 | — | — | — | — | — | — |
| Superior Longitudinal fasc L | 0.28 | 3.402 | 0.911 | 0.63 | -2.968 | -1.061 | — | — | — | 1.02 | -3.337 | -0.727 | — | — | — | 0.31 | -3.909 | -0.805 |
| Superior Longitudinal fasc R | — | — | — | 1.50 | -2.743 | -1.009 | — | — | — | 2.30 | -3.332 | -0.751 | — | — | — | 0.43 | -3.930 | -0.681 |
| Uncinate fasc L | — | — | — | 3.53 | -2.638 | -1.357 | — | — | — | 1.90 | -3.312 | -0.756 | — | — | — | — | — | — |
| Uncinate fasc R | — | — | — | 5.32 | -2.567 | -1.617 | — | — | — | 0.49 | -3.130 | -0.570 | — | — | — | — | — | — |
| Sup Longitudinal fasc temporal L | 0.64 | 3.408 | 0.911 | 0.51 | -2.951 | -1.018 | — | — | — | 1.31 | -3.344 | -0.727 | — | — | — | 0.74 | -3.909 | -0.805 |
| Sup Longitudinal fasc temporal R | — | — | — | 1.85 | -2.878 | -0.989 | — | — | — | 0.14 | -3.575 | -0.427 | — | — | — | — | — | — |
| **ICBM-DTI 81** | Vol (%) | t | max g | Vol (%) | t | max g | Vol (%) | t | max ρ | Vol (%) | t | max ρ | Vol (%) | t | max ρ | Vol (%) | t | max ρ |
| Middle cerebellar peduncle | — | — | — | — | — | — | — | — | — | 2.52 | -3.079 |  | — | — | — | — | — | — |
| Genu of corpus callosum | — | — | — | 4.16 | -2.963 | -1.102 | — | — | — | 0.40 | -2.781 |  | — | — | — | 3.94 | -3.622 | -0.598 |
| Body of corpus callosum | — | — | — | — | — | — | — | — | — | 3.66 | -2.934 | -0.536 | — | — | — | 2.22 | -3.433 | -0.628 |
| Splenium of corpus callosum | — | — | — | — | — | — | — | — | — | 4.93 | -3.106 | -0.630 | — | — | — | 0.27 | -3.409 | -0.721 |
| Medial lemniscus R | — | — | — | — | — | — | — | — | — | 0.14 | -2.767 |  | — | — | — | — | — | — |
| Anterior limb of internal capsule R | — | — | — | 21.61 | -3.976 | -1.121 | 1.40 | 3.405 | 0.635 | — | — | — | — | — | — | — | — | — |
| Anterior limb of internal capsule L | — | — | — | 6.23 | -3.881 | -1.017 | — | — | — | — | — | — | — | — | — | — | — | — |
| Posterior limb of internal capsule R | — | — | — | 6.23 | -3.761 | -1.119 | 4.34 | 3.201 | 0.606 | — | — | — | 3.36 | 3.332 | 0.721 | — | — | — |
| Retrolenticular part of internal capsule R | — | — | — | 0.12 | -2.187 | -0.605 | — | — | — | — | — | — | — | — | — | — | — | — |
| Anterior corona radiata R | — | — | — | 8.02 | -3.287 | -1.165 | — | — | — | 0.06 | -3.128 |  | — | — | — | 0.96 | -3.299 | -0.600 |
| Anterior corona radiata L | — | — | — | 3.36 | -3.100 | -1.300 | — | — | — | 2.69 | -2.982 | -0.592 | — | — | — | — | — | — |
| Superior corona radiata R | — | — | — | 11.67 | -3.622 | -0.949 | — | — | — | 5.33 | -3.263 | -0.646 | — | — | — | 1.92 | -3.309 | -0.741 |
| Posterior corona radiata R | — | — | — | 1.90 | -3.323 | -1.080 | — | — | — | — | — | — | — | — | — | 10.06 | -3.620 | -0.746 |
| Posterior corona radiata L | — | — | — | — | — | — | — | — | — | 1.43 | -2.738 |  | — | — | — | — | — | — |
| Posterior thalamic radiation R | 1.36 | 3.070 | 1.096 | — | — | — | — | — | — | — | — | — | — | — | — | — | — | — |
| Posterior thalamic radiation L | — | — | — | 1.91 | -3.166 | -1.177 | — | — | — | 8.85 | -3.111 |  | — | — | — | — | — | — |
| Sagittal stratum L | 6.10 | 3.258 | 0.912 | 0.13 | -2.600 | -0.917 | — | — | — | — | — | — | — | — | — | — | — | — |
| External capsule R | — | — | — | 8.84 | -3.590 | -1.170 | — | — | — | — | — | — | — | — | — | — | — | — |
| External capsule L | — | — | — | 0.14 | -2.200 | -1.298 | — | — | — | — | — | — | — | — | — | — | — | — |
| Cingulum (cingulate gyrus) R | — | — | — | — | — | — | — | — | — | 0.47 | -3.149 |  | — | — | — | — | — | — |
| Fornix (cres) / Stria terminalis L | — | — | — | 0.62 | -2.486 | -0.665 | — | — | — | — | — | — | — | — | — | — | — | — |
| Superior longitudinal fasciculus R | — | — | — | 0.59 | -2.552 | -1.009 | — | — | — | — | — | — | — | — | — | — | — | — |
| Superior longitudinal fasciculus L | — | — | — | 1.20 | -3.669 | -1.005 | — | — | — | 0.74 | -3.159 | -0.458 | — | — | — | 2.06 | -3.909 | -0.805 |
| Superior fronto-occipital fasciculus R | — | — | — | 14.00 | -3.150 | -0.812 | — | — | — | — | — | — | — | — | — | — | — | — |
| Uncinate fasciculus L | — | — | — | 2.66 | -2.528 | -0.804 | — | — | — | — | — | — | — | — | — | — | — | — |
| Tapetum R | 6.04 | 2.935 | 1.018 | — | — | — | — | — | — | — | — | — | — | — | — | — | — | — |
| **NODDI: NDI** | | | | | | | | | |  |  |  |  |  |  |  |  |  |
|  | **NDI** | | | **NDI - MoCA correlation** | | | **NDI - GOS-E correlation** | | |  |  |  |  |  |  |  |  |  |
|  | HC > Concussion | | | t > 0 | | | t > 0 | | |  |  |  |  |  |  |  |  |  |
| **JHU white matter** | Vol (%) | t | max g | Vol (%) | t | max ρ | Vol (%) | t | max ρ |  |  |  |  |  |  |  |  |  |
| Anterior Thalamic Radiation L | 1.27 | 3.116 | 0.699 | 0.92 | 2.781 | 0.622 | 1.92 | 3.372 | 0.828 |  |  |  |  |  |  |  |  |  |
| Anterior Thalamic Radiation R | — | — | — | 0.51 | 2.562 | 0.549 | 2.53 | 3.327 | 0.769 |  |  |  |  |  |  |  |  |  |
| Cortical spinal tract L | — | — | — | — | — | — | 0.57 | 3.314 | 0.690 |  |  |  |  |  |  |  |  |  |
| Cingulum cingulate gyrus L | 0.49 | 2.803 | 0.748 | 6.19 | 2.688 | 0.649 | — | — | — |  |  |  |  |  |  |  |  |  |
| Cingulum cingulate gyrus R | — | — | — | 0.42 | 2.546 | 0.590 | — | — | — |  |  |  |  |  |  |  |  |  |
| Forceps Major | — | — | — | 0.88 | 3.093 | 0.706 | — | — | — |  |  |  |  |  |  |  |  |  |
| Forceps Minor | 0.24 | 3.026 | 0.646 | 9.05 | 2.819 | 0.707 | — | — | — |  |  |  |  |  |  |  |  |  |
| Inferior fronto-occipital fasc L | 0.84 | 3.057 | 0.699 | 0.13 | 2.637 | 0.601 | — | — | — |  |  |  |  |  |  |  |  |  |
| Inferior fronto-occipital fasc R | — | — | — | 0.32 | 2.800 | 0.684 | — | — | — |  |  |  |  |  |  |  |  |  |
| Inferior Longitudinal fasc L | — | — | — | — | — | — | 0.18 | 3.346 | 0.582 |  |  |  |  |  |  |  |  |  |
| Inferior Longitudinal fasc R | 0.46 | 2.988 | 1.115 | — | — | — | — | — | — |  |  |  |  |  |  |  |  |  |
| Superior Longitudinal fasc R | 0.35 | 3.018 | 1.067 | — | — | — | 0.33 | 4.009 | 0.698 |  |  |  |  |  |  |  |  |  |
| Uncinate fasc L | 0.72 | 2.821 | 0.536 | 0.17 | 2.625 | 0.530 | — | — | — |  |  |  |  |  |  |  |  |  |
| Uncinate fasc R | 0.68 | 2.977 | 0.663 | 0.10 | 2.405 | 0.437 | — | — | — |  |  |  |  |  |  |  |  |  |
| **ICBM-DTI 81** | Vol (%) | t | max g | Vol (%) | t | max ρ | Vol (%) | t | max ρ |  |  |  |  |  |  |  |  |  |
| Genu of corpus callosum | — | — | — | 30.37 | 2.893 | 0.707 | — | — | — |  |  |  |  |  |  |  |  |  |
| Body of corpus callosum | — | — | — | 4.78 | 2.876 | 0.623 | — | — | — |  |  |  |  |  |  |  |  |  |
| Splenium of corpus callosum | — | — | — | 9.00 | 3.202 | 0.726 | — | — | — |  |  |  |  |  |  |  |  |  |
| Corticospinal tract L | — | — | — | — | — | — | 0.44 | 3.200 | 0.490 |  |  |  |  |  |  |  |  |  |
| Cerebral peduncle L | — | — | — | — | — | — | 2.99 | 3.333 | 0.690 |  |  |  |  |  |  |  |  |  |
| Anterior limb of internal capsule R | — | — | — | — | — | — | 12.24 | 3.309 | 0.745 |  |  |  |  |  |  |  |  |  |
| Anterior limb of internal capsule L | — | — | — | — | — | — | 1.69 | 3.603 | 0.817 |  |  |  |  |  |  |  |  |  |
| Posterior limb of internal capsule R | — | — | — | — | — | — | 4.66 | 3.461 | 0.769 |  |  |  |  |  |  |  |  |  |
| Posterior limb of internal capsule L | — | — | — | — | — | — | 5.36 | 3.127 | 0.630 |  |  |  |  |  |  |  |  |  |
| Retrolenticular part of internal capsule L | — | — | — | — | — | — | 2.67 | 3.361 | 0.611 |  |  |  |  |  |  |  |  |  |
| Anterior corona radiata R | — | — | — | 4.95 | 2.671 | 0.614 | — | — | — |  |  |  |  |  |  |  |  |  |
| Anterior corona radiata L | 0.39 | 2.572 | 0.571 | 7.69 | 2.587 | 0.547 | — | — | — |  |  |  |  |  |  |  |  |  |
| Posterior corona radiata R | — | — | — | 0.78 | 2.965 | 0.654 | — | — | — |  |  |  |  |  |  |  |  |  |
| Posterior thalamic radiation R | — | — | — | 0.15 | 2.907 | 0.658 | — | — | — |  |  |  |  |  |  |  |  |  |
| Cingulum (cingulate gyrus) R | — | — | — | 0.26 | 2.524 | 0.634 | — | — | — |  |  |  |  |  |  |  |  |  |
| Superior fronto-occipital fasciculus L | — | — | — | — | — | — | 11.44 | 3.819 | 0.557 |  |  |  |  |  |  |  |  |  |

**Supplementary Table 3**

Voxel-wise group comparisons and clinical correlations with MoCA and GOS-E scores for NODDI metrics. The table presents the complete results for FWF, ODI, and NDI based on the Johns Hopkins University (JHU) white matter atlas and the ICBM-DTI-81 atlas. For each significant cluster (FDR < 0.05), the percentage of the white matter ROI volume (Vol %), mean t-value, and maximum effect-sizes (max g or max ρ) are reported.
